# MafB-restricted local monocyte proliferation precedes lung interstitial macrophage differentiation

**DOI:** 10.1038/s41590-023-01468-3

**Published:** 2023-03-16

**Authors:** Domien Vanneste, Qiang Bai, Shakir Hasan, Wen Peng, Dimitri Pirottin, Joey Schyns, Pauline Maréchal, Cecilia Ruscitti, Margot Meunier, Zhaoyuan Liu, Céline Legrand, Laurence Fievez, Florent Ginhoux, Coraline Radermecker, Fabrice Bureau, Thomas Marichal

**Affiliations:** 1 Laboratory of Immunophysiology, GIGA Institute, Liège University, Liège, Belgium; 2 Faculty of Veterinary Medicine, Liège University, Liège, Belgium; 3 Laboratory of Cellular and Molecular Immunology, GIGA Institute, Liège University, Liège, Belgium; 4 Shanghai Institute of Immunology, Shanghai JiaoTong University School of Medicine, Shanghai, China; 5 Inserm U1015, Gustave Roussy, Bâtiment de Médecine Moléculaire, Villejuif, France; 6 Singapore Immunology Network (SIgN), Agency for Science, Technology and Research (A*STAR), Singapore, Singapore; 7 Translational Immunology Institute, SingHealth Duke-NUS Academic Medical Centre, Singapore, Singapore; 8 Walloon Excellence in Life Sciences and Biotechnology (WELBIO) Department, WEL Research Institute, Wavre, Belgium; 9 Present Address: Department of Immunology, University of Toronto, Toronto, Canada

**Keywords:** Monocytes and macrophages, Cell biology

## Abstract

Resident tissue macrophages (RTMs) are differentiated immune cells that populate distinct niches and exert important tissue-supportive functions. RTM maintenance is thought to rely either on differentiation from monocytes or on RTM self-renewal. Here, we used a mouse model of inducible lung interstitial macrophage (IM) niche depletion and refilling to investigate the development of IMs in vivo. Using time-course single-cell RNA-sequencing analyses, bone marrow chimeras and gene targeting, we found that engrafted Ly6C^+^ classical monocytes proliferated locally in a Csf1 receptor-dependent manner before differentiating into IMs. The transition from monocyte proliferation toward IM subset specification was controlled by the transcription factor MafB, while c-Maf specifically regulated the identity of the CD206^+^ IM subset. Our data provide evidence that, in the mononuclear phagocyte system, the ability to proliferate is not merely restricted to myeloid progenitor cells and mature RTMs but is also a tightly regulated capability of monocytes developing into RTMs in vivo.

## Abstract (web summary)

Marichal and colleagues use a model of niche depletion and refilling to show that engrafted Ly6C^+^ classical monocytes proliferate locally in a Csf1 receptor-dependent manner before differentiating into lung interstitial macrophages.

## Main

RTMs are self-maintaining immune cells that are integral parts of mammalian tissues and exert important tissue-supportive functions. The original understanding that RTMs arise from bone marrow (BM)-derived circulating monocytes, as proposed by van Furth and Cohn^1^, has been challenged by multiple reports showing that several RTM populations can arise from embryonic yolk sac macrophages and fetal monocytes that seed the tissues before the establishment of definitive hematopoiesis, and can self-maintain with minimal contribution of monocytes^2–5^. Nevertheless, throughout adult life, monocytes can give rise to RTMs in proportions that depend on the tissue accessibility and on the nature and extent of perturbations leading to RTM depletion^6–10^.

Besides origin, the differentiation trajectories and the tissue cues are thought to be essential determinants of RTM identity and function^6,11,12^. In a given niche, RTMs can respond to local trophic factors, such as Csf1 for their maintenance, and are instructed by niche-derived signals that trigger the expression of specific transcription factors and differentiation programs, thereby tailoring a specific identity that fulfills the functional needs of a given tissue^6,13–15^.

While currently the repopulation and maintenance of RTM niches is thought to be achieved either through monocyte engraftment and differentiation or through the self-renewal of mature RTMs^8,11^, the slow turnover of RTMs at steady state and the lack of models that allow the capture of rare events, such as monocyte-to-RTM transitioning cells, have hampered investigations of RTM dynamics in vivo. The lung IMs, which are long-lived RTMs that are slowly replenished in adults by Ly6C^+^ classical monocytes (cMo) and encompass perivascular CD206^+^ IMs and nerve-associated CD206^−^ IMs^16–20^, can be used as a model to study monocyte-to-RTM trajectories. Here, we developed a transgenic mouse model of diphtheria toxin (DT)-inducible IM niche depletion that allowed us to capture and explore at the single-cell resolution the dynamics of events that occur during monocyte-to-IM differentiation. In this model, we found that repopulated IMs arose from BM-derived Ly6C^+^ cMo dependent on the monocyte chemoattractant receptor Ccr2 that could undergo a transient Csf1 receptor (Csf1r)-dependent proliferation in vacant tissue niches before their differentiation into CD206^+^ IMs or CD206^−^ IMs, a process that was regulated by MafB. Our data support the idea that tissue monocyte proliferation might represent an underappreciated process involved in monocyte-to-RTM trajectories in vivo.

## Results

### Lung interstitial macrophages express high levels of *Tmem119* and *Cx3cr1*

**Fig. 1:**
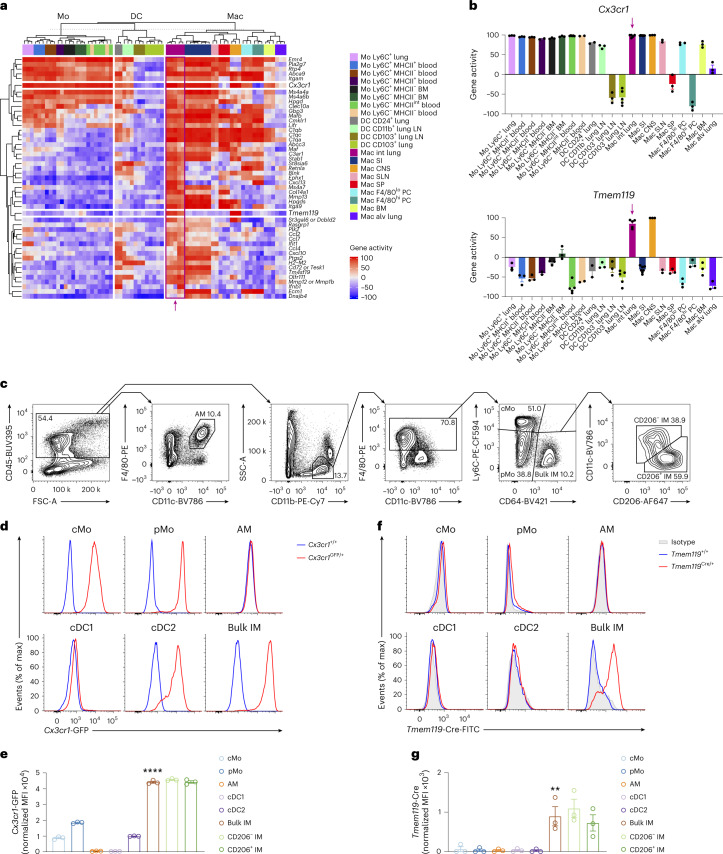
Lung interstitial macrophage subsets can be defined as *Cx3cr1*^hi^*Tmem119*^hi^ cells. **a**, Heat map showing gene activity in the indicated myeloid cell populations, inferred from microarray data uploaded on the Gene Expression Commons platform. Alv, alveolar; CNS, central nervous system; Int, interstitial; LN, lymph node; Mo, monocyte; Mac, macrophage; PC, peritoneal cavity; SI, small intestine; SLNs, skin-draining lymph nodes; SP, spleen. **b**, Gene activities of *Cx3cr1* and *Tmem119* in the indicated myeloid cell populations, as in **a**. The arrow indicates lung IMs. **c**, Representative flow cytometry gating strategy showing CD45^+^F4/80^+^CD11c^+^ AMs, AM-excluded CD45^+^SSC^lo^CD11b^+^F4/80^+^Ly6C^+^CD64^−^ cMo, AM-excluded CD45^+^SSC^lo^CD11b^+^F4/80^+^Ly6C^−^CD64^−^ pMo, AM-excluded CD45^+^SSC^lo^CD11b^+^F4/80^+^CD64^+^ bulk IMs further divided into CD206^+^ IMs and CD206^−^ IMs in lungs of wild-type mice at steady state. **d**,**e**, Representative flow cytometry histograms (**d**) and normalized MFI (**e**) of GFP expression in lung cMo, pMo, AMs and IMs, as in **c**, and in CD45^+^CD11c^+^MHC-II^+^CD26^+^CD64^−^CD172a^−^XCR1^+^ type 1 conventional DCs (cDC1) and CD45^+^CD11c^+^MHC-II^+^CD26^+^CD64^−^CD172a^+^MAR1^−^ (cDC2) from *Cx3cr1*^GFP/+^ and *Cx3cr1*^+/+^ mice. **f**,**g**, Representative histograms (**f**) and normalized MFI (**g**) of intracellular Cre protein in lung myeloid cells, as in **d** and **e**, from *Tmem119*^Cre/+^ and *Tmem119*^+/+^ mice. Data show the mean ± s.e.m. and individual values (**b**, **e** and **g**: *n* = 3 replicates, 3 mice and 3 mice, respectively). *P* values were calculated using a one-way analysis of variance (ANOVA) with Tukey’s post hoc test and compared bulk IMs with cMo, pMo, AMs, cDC1 and cDC2 (**e** and **g**). ^**^P < 0.01; ^****^P < 0.0001. MFI, mean fluorescence intensity. Source data

**Extended Data Fig. 1:**
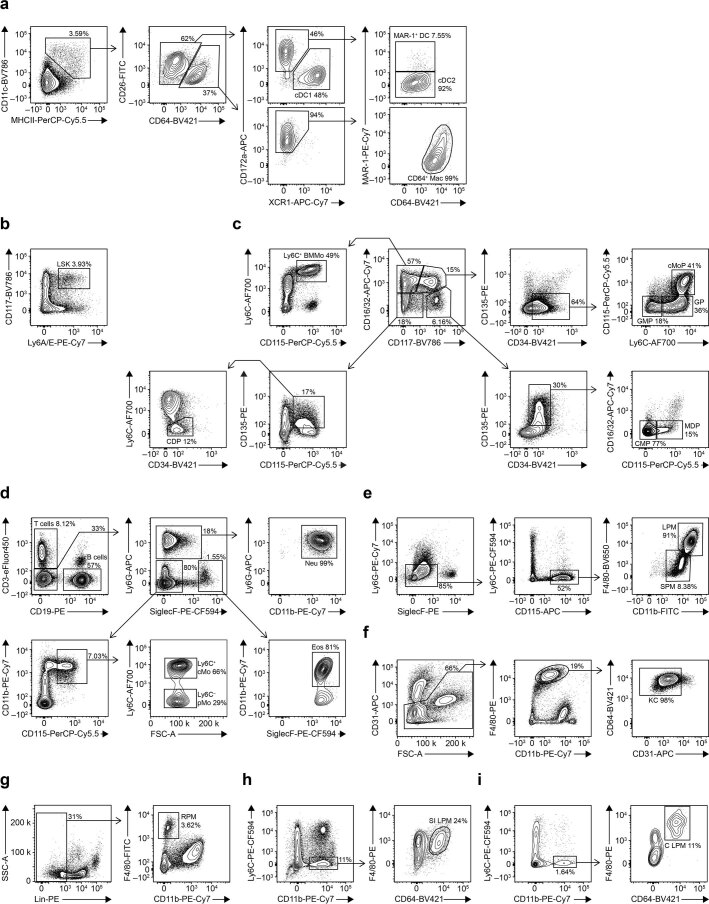
Flow cytometry gating strategies to delineate lung DCs, BM progenitors, blood immune cells and RTM. Flow cytometry gating strategy used to delineate lung CD45^+^CD11c^+^MHC-II^+^CD26^+^CD64^−^CD172a^−^XCR1^+^ type 1 conventional DC (cDC1), CD45^+^CD11c^+^MHC-II^+^CD26^+^CD64^−^CD172a^+^MAR1^−^ type 2 conventional DC (cDC2), CD45^+^CD11c^+^MHC-II^+^CD26^+^CD64^−^CD172a^+^MAR1^+^ DCs (MAR1^+^ DC) and CD45^+^CD11c^+^MHC-II^+^CD26^−^CD64^+^CD172a^+^ macrophages (CD64^+^ Mac) (**a**), BM Lin^−^Ly6A/E^+^CD117^+^ LSK (**b**), Lin^−^CD16/32^−^CD117^+^CD135^+^CD34^+^CD115^−^ common myeloid progenitors (CMP), Lin^−^CD16/32^−^CD117^+^CD135^+^CD34^+^CD115^+^ monocyte-DC progenitors (MDP), Lin^−^CD16/32^+^CD117^+^CD135^−^CD34^+^CD115^−^Ly6C^−^ granulocyte-monocyte progenitors (GMP), Lin^−^CD16/32^+^CD117^+^CD135^−^CD34^+^CD115^−^Ly6C^+^ granulocyte progenitors (GP), Lin^−^CD16/32^+^CD117^+^CD135^−^CD34^+^CD115^+^Ly6C^+^ monocyte progenitors (cMoP), Lin-CD16/32^+^CD117^−^CD115^+^Ly6C^+^ monocytes (Ly6C^+^ BMMo), Lin^−^CD16/32^−^CD117^−^CD135^+^CD115^+^CD34^−^Ly6C^−^ common DC progenitors (CDP) (**c**), blood CD45^+^CD3^+^CD19^−^ T cells, CD45^+^CD3^−^CD19^+^ B cells, CD45^+^CD3^−^CD19^−^Ly6G^−^SiglecF^−^CD115^+^ Ly6C^+^ cMo or Ly6C^−^ pMo, CD45^+^CD3^−^CD19^−^Ly6G^+^CD11b^+^ neutrophils (Neu), CD45^+^CD3^−^CD19^−^CD11b^+^SiglecF^+^ eosinophils (Eos) (**d**), CD45^+^Ly6G^−^SiglecF^−^Ly6C^−^CD115^+^CD11b^+^ F4/80^hi^ large (LPM) or F4/80^lo^ small peritoneal macrophages (SPM) (**e**), liver CD45^+^CD31^−^F4/80^+^CD11b^int^CD64^+^ Kupffer cells (KC) (**f**), spleen Lin^−^F4/80^+^CD11b^−^ red pulp macrophages (RPM) (**g**), small intestinal CD45^+^Ly6C^−^CD11b^+^F4/80^+^CD64^+^ lamina propria macrophages (SI LPM) (**h**) and colon CD45^+^Ly6C^−^CD11b^+^F4/80^+^CD64^+^ lamina propria macrophages (C LPM) (**i**). Mac, macrophage.

**Extended Data Fig. 2:**
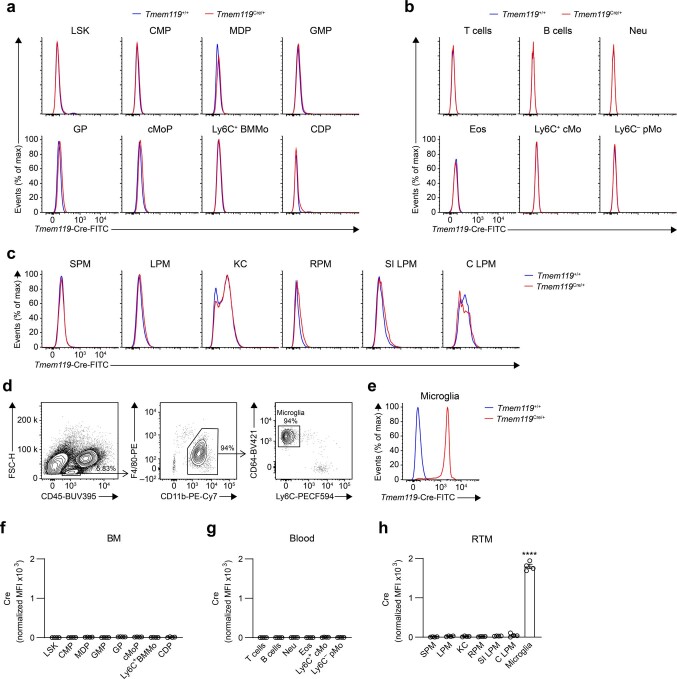
Assessment of intracellular Cre protein expression in BM progenitors, blood leukocytes and RTM. **a-c**, Representative histograms of Cre intracellular expression in BM progenitors, as in Extended Data Fig. 1b,c (**a**), in blood leukocytes, as in Extended Data Fig. 1d (**b**), and in small and large peritoneal macrophages (SPM and LPM, respectively), Kupffer cells (KC), red pulp splenic macrophages (RPM), small intestinal (SI) and colon (C) lamina propria macrophages (LPM), as in Extended Data Fig. 1e-i (**c**) from *Tmem119*^+/+^ (blue) and *Tmem119*^Cre/+^ (red) mice. **d**, Flow cytometry gating strategy to show brain FSC^lo^CD45^int^F4/80^+^CD11b^+^CD64^+^Ly6C^−^ microglia. **e**, Representative histograms of Cre intracellular protein expression in microglia from *Tmem119*^+/+^ (blue) and *Tmem119*^Cre/+^ (red) mice. **f-h**, Bar graphs showing normalized Cre expression in the indicated cell populations from the BM (**f**) and the blood (**g**), and in tissue RTM (**h**). Data show mean ± SEM (*n* = 4 mice per group). In **h**, *P* values compare microglia vs. every other population and were calculated using a one-way analysis of variance (ANOVA) with Tukey’s post hoc tests. Raw data and *P* values are provided as a source data file. ^****^, *P* < 0.0001. CDP, common plasmacytoid and dendritic cell progenitor; cMoP, common monocyte progenitor; CMP, common myeloid progenitor; GMP, granulocyte-monocyte progenitor; GP, granulocyte progenitor; LSK, lineage(Lin)^−^Sca-1^+^cKit^+^ multipotent progenitor; MDP, monocyte-dendritic cell progenitor. Source data

**Extended Data Fig. 3:**
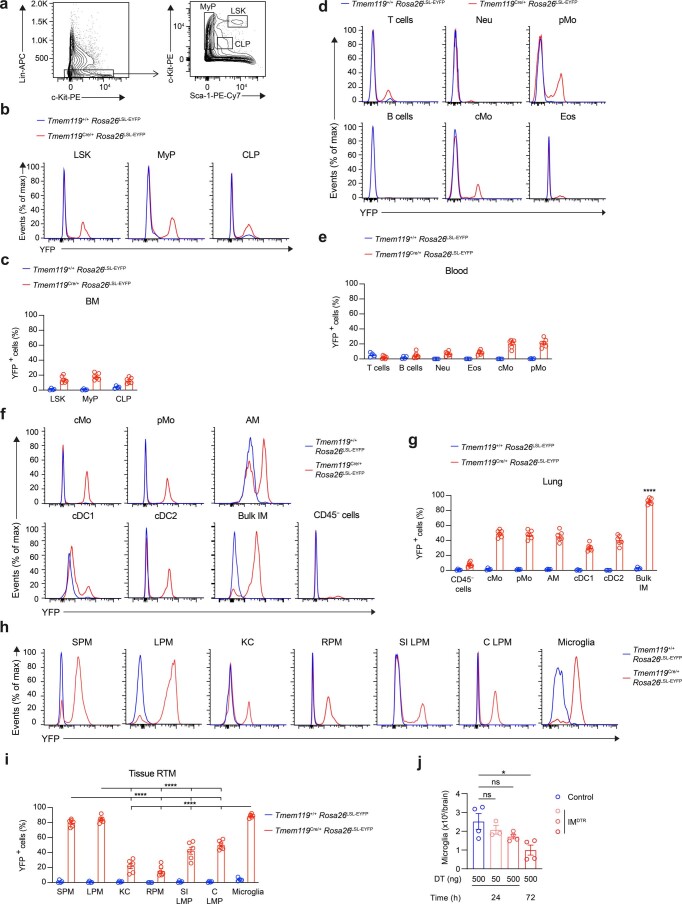
Assessment of YFP labeling in *Tmem119*^Cre^*Rosa26*^LSL-EYFP^ mice and of microglia depletion in IM^DTR^ mice. **a**, Flow cytometry gating strategy to show Lineage(Lin)^−^Sca-1^+^c-Kit^+^ (LSK) multipotent progenitors, Lin^−^Sca-1^−^c-Kit^int/hi^ myeloid lineage-committed progenitors (MyP) and Lin^−^Sca-1^int^c-Kit^int^ common lymphoid progenitors (CLP). **b-c**, Representative histograms of YFP expression (**b**) and bar graphs showing % of YFP^+^ cells (**c**) in BM LSK, MyP and CLP (**c**) from *Tmem119*^*+/+*^*Rosa26*^LSL-EYFP^ (blue) and *Tmem119*^Cre/+^*Rosa26* ^LSL-EYFP^ (red) mice. **d-e**, Representative histograms of YFP expression (**d**) and bar graphs showing % of YFP^+^ cells (**e**) in blood CD45^+^CD3^+^CD19^−^ T cells, CD45^+^CD3^−^CD19^+^ B cells, CD45^+^CD3^−^CD19^−^Ly6G^−^SiglecF^−^CD115^+^ Ly6C^+^ cMo or Ly6C^−^ pMo, CD45^+^CD3^−^CD19^−^Ly6G^+^CD11b^+^ neutrophils (Neu), CD45^+^CD3^−^CD19^−^CD11b^+^SiglecF^+^ eosinophils (Eos) from *Tmem119*^*+/+*^*Rosa26*^LSL-EYFP^ (blue) and *Tmem119*^Cre/+^*Rosa26* ^LSL-EYFP^ (red) mice. **f-g**, Representative histograms of YFP expression (**f**) and bar graphs showing % of YFP^+^ cells (**g**) in lung cMo, pMo, cDC1, cDC2, AMs, IMs and CD45^−^ structural cells from *Tmem119*^*+/+*^*Rosa26*^LSL-EYFP^ (blue) and *Tmem119*^Cre/+^*Rosa26* ^LSL-EYFP^ (red) mice. **h-i**, Representative histograms of YFP expression (**h**) and bar graphs showing % of YFP^+^ cells (**i**) in small and large peritoneal macrophages (SPM and LPM, respectively), Kupffer cells (KC), red pulp splenic macrophages (RPM), small intestinal (SI) and colon (C) lamina propria macrophages (LPM) and microglia from *Tmem119*^*+/+*^*Rosa26*^LSL-EYFP^ (blue) and *Tmem119*^Cre/+^*Rosa26* ^LSL-EYFP^ (red) mice. **j**, Numbers of microglia quantified by flow cytometry in IM^DTR^ and littermate control mice injected i.p. with 50 or 500 ng DT and evaluated 24 or 72 h post-DT injection. Data show mean ± SEM and are pooled from 2 independent experiments (**c,e,g,i,j**) (c,e,g,i: *Tmem119*^*+/+*^*Rosa26*^LSL-EYFP^; *Tmem119*^Cre/+^*Rosa26* ^LSL-EYFP^: *n* = 4;6 mice per group, respectively; j: *n* = 3-4 mice per group). *P* values were calculated using a two-way (**c,e,g,i**) or a one-way (**j**) analysis of variance (ANOVA) with Tukey’s post hoc test. In **g**, *P* values compare IMs vs. every other population. Raw data and *P* values are provided as a source data file. ^*^, *P* < 0.05; ^****^, *P* < 0.0001. ns, not significant. Source data

We uploaded microarray data from the ImmGen database^21^ and published datasets^18^ into the Gene Expression Commons platform^22^ and found that IMs, as well as microglia, had high expression of the genes encoding the fractalkine receptor (*Cx3cr1*) and the transmembrane protein 119 (*Tmem119*; Fig. 1a,b). Flow cytometry of myeloid cells isolated from the lungs of *Cx3cr1*^GFP/+^ mice indicated that lung CD45^+^SSC^lo^CD11b^+^F4/80^+^CD64^+^ IMs (called IMs hereafter) expressed high levels of GFP (Fig. 1c–e and Extended Data Fig. 1a). Next, we used CRISPR/Cas9-mediated engineering to generate C57BL/6 mice expressing Cre recombinase under the control of endogenous *Tmem119* (hereafter *Tmem119*^Cre^ mice). Quantification of intracellular expression of Cre protein by flow cytometry indicated elevated Cre expression in the CD206^−^ IM and CD206^+^ IM subsets, but no detectable Cre in other lung myeloid cells (Fig. 1f,g), BM progenitors, blood leukocytes and RTMs in the peritoneum, liver, spleen and gut, with the exception of microglia (Extended Data Fig. 1b–i and Extended Data Fig. 2) in *Tmem119*^Cre^ mice. We crossed *Tmem119*^Cre^ mice with the *Rosa26*^LSL-EYFP^ reporter strain^23^, resulting in *Tmem119*^Cre^*Rosa26*^LSL-EYFP^ mice in which persistent enhanced YFP protein expression is induced in Tmem119-expressing cells and their progeny. Less than 25% of multipotent, myeloid lineage-committed and common lymphoid progenitors in the BM of *Tmem119*^Cre^*Rosa26*^LSL-EYFP^ mice were YFP^+^ (Extended Data Fig. 3a–c). B cells, T cells, neutrophils and eosinophils in the blood exhibited almost no YFP labeling, while 10–30% of cMo and Ly6C^−^ patrolling monocytes (pMo) were YFP^+^ (Extended Data Fig. 3d,e). While lung CD45^−^ structural cells exhibited very low YFP expression, ~50% of lung cMo, pMo, alveolar macrophages (AMs) and dendritic cells (DCs) were YFP^+^ (Extended Data Fig. 3f,g). IMs had the highest YFP labeling (92%) among all lung myeloid cell populations tested (Extended Data Fig. 3f,g). RTMs in other tissues exhibited variable expression of YFP, with microglia (89%) and small (80%) and large (84%) peritoneal macrophages displaying a similar pattern as lung IMs (Extended Data Fig. 3h,i). Combined with the Cre staining, these results indicated that IMs actively expressed Cre, while YFP labeling observed in lung monocytes, DCs and other RTMs, except microglia, reflected a history of transient *Tmem119* expression in progenitor cells.

### Classical monocytes give rise to interstitial macrophage subsets upon niche depletion

**Fig. 2:**
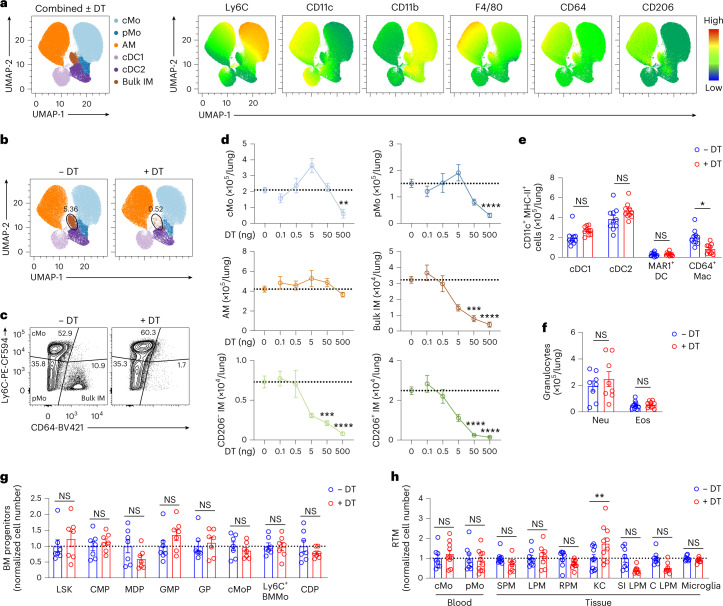
Efficiency and specificity of diphtheria toxin-induced interstitial macrophage depletion in IM^DTR^ mice. **a**, Representative merged UMAP plots of lung single live CD45^+^CD11b^+^ or CD11c^+^ mononuclear cells analyzed by flow cytometry 24 h after 50 ng DT i.p. injection or no treatment in IM^DTR^ mice (merged data from four mice per group). Cell clusters (left) and heat map plots depicting the expression of Ly6C, CD11c, CD11b, F4/80, CD64 and CD206 (right). **b**, Representative UMAP plot, as in **a**, showing cells from either untreated IM^DTR^ mice or DT-treated IM^DTR^ mice. **c**, Representative contour plot of Ly6C and CD64 expression within lung single live AM-excluded CD45^+^SSC^lo^CD11b^+^F4/80^+^ cells from untreated and DT-treated IM^DTR^ mice, as in **a**. **d**, Absolute numbers of the indicated lung myeloid cell populations quantified by flow cytometry in IM^DTR^ mice, at 24 h after i.p. injection with DT in doses ranging from 0.1 to 500 ng. Horizontal dotted lines represent the average number of cells in untreated IM^DTR^ mice. **e**,**f**, Absolute numbers of lung CD45^+^CD11c^+^MHC-II^+^CD26^+^CD64^−^CD172a^−^XCR1^+^ cDC1, CD45^+^CD11c^+^MHC-II^+^CD26^+^CD64^−^CD172a^+^MAR1^−^ cDC2, CD45^+^CD11c^+^MHC-II^+^CD26^+^CD64^−^CD172a^+^MAR1^+^ DCs (MAR1^+^ DC) and CD45^+^CD11c^+^MHC-II^+^CD26^−^CD64^+^CD172a^+^ macrophages (CD64^+^ Mac) (**e**) and lung CD45^+^CD11b^+^Ly6G^+^ neutrophils (Neu) and CD45^+^CD11b^+^SiglecF^+^ eosinophils (Eos) (**f**) quantified by flow cytometry 24 h after 50 ng DT i.p. injection or no treatment in IM^DTR^ mice. **g**,**h**, Numbers of BM Lin^−^Ly6A/E^+^CD117^+^ LSK, Lin^−^CD16/32^−^CD117^+^CD135^+^CD34^+^CD115^−^ common myeloid progenitors (CMP), Lin^−^CD16/32^−^CD117^+^CD135^+^CD34^+^CD115^+^ monocyte-DC progenitors (MDP), Lin^−^CD16/32^+^CD117^+^CD135^−^CD34^+^CD115^−^Ly6C^−^ granulocyte-monocyte progenitors (GMP), Lin^−^CD16/32^+^CD117^+^CD135^−^CD34^+^CD115^−^Ly6C^+^ granulocyte progenitors (GP), Lin^−^CD16/32^+^CD117^+^CD135^−^CD34^+^CD115^+^Ly6C^+^ monocyte progenitors (cMoP), Lin^−^CD16/32^+^CD117^−^CD115^+^Ly6C^+^ monocytes (Ly6C^+^ BMMo), Lin^−^CD16/32^−^CD117^−^CD135^+^CD115^+^CD34^−^Ly6C^−^ common DC progenitors (CDP) (**g**), blood CD45^+^CD3^−^CD19^−^Ly6G^−^SiglecF^−^CD115^+^ Ly6C^+^ cMo or Ly6C^−^ pMo, CD45^+^Ly6G^−^SiglecF^−^Ly6C^−^CD115^+^CD11b^+^ F4/80^hi^ large (LPM) or F4/80^lo^ small peritoneal macrophages (SPM), liver CD45^+^CD31^−^F4/80^+^CD11b^int^CD64^+^ Kupffer cells (KC), spleen Lin^−^F4/80^+^CD11b^−^ red pulp macrophages (RPM), small intestinal (SI) and colon (C) CD45^+^Ly6C^−^CD11b^+^F4/80^+^CD64^+^ lamina propria macrophages (LPM) and FSC^lo^CD45^int^F4/80^+^CD11b^+^CD64^+^Ly6C^−^ microglia (**h**), as in **e**. Data show the mean ± s.e.m. and are pooled from 2–4 independent experiments (**d**–**h**: *n* = 6–15, 10, 8–10, 7, 8–10 mice per group, respectively). *P* values were calculated using a two-way ANOVA with Tukey’s post hoc test. ^*^P < 0.05; ^**^P < 0.01; ^***^P < 0.001; ^****^P < 0.0001; NS, not significant. Source data

Like for most RTM populations, monocyte engraftment and differentiation into IMs are rare events at steady state^18,20^. Hence, to investigate the dynamics of IM development in vivo, we sought to accelerate this process by creating a vacant niche that would presumably be rapidly refilled, as shown for other RTMs^11,24^. To this end, we generated a transgenic model of DT-induced lung IM depletion by crossing *Tmem119*^Cre^ and *Cx3cr1*^LSL-DTR^ mice^25^. In *Tmem119*^Cre^*Cx3cr1*^LSL-DTR^ mice, referred to as IM^DTR^ mice hereafter, cells that express both *Cx3cr1* and *Tmem119* should express the diphtheria toxin receptor (DTR) and be sensitive to DT-induced death. Intraperitoneal (i.p.) injection of IM^DTR^ mice with 50 ng DT led to the efficient depletion of both CD206^+^ IM and CD206^−^ IM subsets 24 h after injection compared with untreated IM^DTR^ mice, while lung AMs, cMo and DC subsets were not affected (Fig. 2a–e). Injections (i.p.) with DT in doses ranging from 0.1 to 500 ng showed a dose-dependent depletion of IMs in IM^DTR^ mice 24 h after injection, but also a partial depletion of lung cMo and pMo at the highest dose (Fig. 2d). Of note, 50 ng DT i.p. did not trigger the recruitment of lung eosinophils or neutrophils at 24 h after injection (Fig. 2f), indicating that the DT-mediated death of IMs did not trigger overt inflammation. We found no significant effect of 50 ng DT on the numbers of BM progenitors, blood monocytes, microglia and RTMs in the peritoneum, spleen and gut, except for an increase in numbers of Kupffer cells in the liver 24 h after injection in IM^DTR^ mice compared to untreated counterparts (Fig. 2g,h). Of note, 500 ng DT triggered a significant depletion of microglia 72 h after DT in IM^DTR^ mice as compared to controls (Extended Data Fig. 3j). As such, both IM subsets were specifically depleted by 50 ng DT i.p. in IM^DTR^ mice.

**Fig. 3:**
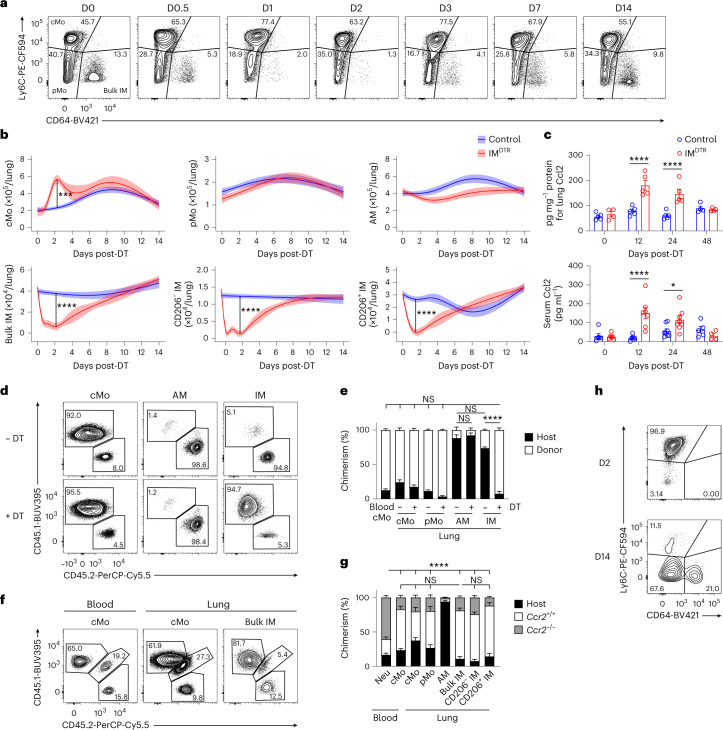
A vacant interstitial macrophage niche is repopulated by Ccr2-dependent classical monocyte differentiating into interstitial macrophages. **a**, Representative plots of Ly6C and CD64 expression within lung AM-excluded CD45^+^F4/80^+^SSC^lo^CD11b^+^ cells at days 0, 0.5, 1, 2, 3, 7 and 14 after 50 ng DT i.p. in IM^DTR^ mice. **b**, Time course of absolute numbers of cMo, pMo, AMs, bulk IMs, CD206^−^ IMs and CD206^+^ IMs quantified by flow cytometry in IM^DTR^ and littermate controls, as in **a**. Data show the mean (centerline) ± s.e.m. (colored area) and are pooled from ≥2 independent experiments (*n* = 8–10 mice per time point). **c**, Amount of Ccl2 in the lung and serum of IM^DTR^ and littermate controls at 0, 12, 24 and 48 h after DT i.p. injection. **d**,**e**, Representative CD45.1 and CD45.2 contour plots (**d**) and bar graphs showing the percentage of CD45.1 donor and CD45.2 host chimerism (**e**) in the indicated cell populations from lethally irradiated thorax-protected CD45.2 IM^DTR^ mice reconstituted with CD45.1 wild-type BM donor cells, injected or not with 50 ng DT i.p. 4 weeks later and evaluated at day 7 after DT. **f**,**g**, Representative CD45.1 and CD45.2 contour plots (**f**) and bar graphs showing the percentage of *Ccr2*^+/+^ donor, *Ccr2*^−/−^ donor and host chimerism (**g**) in the indicated cell populations from lethally irradiated, thorax-protected CD45.1/CD45.2 IM^DTR^ mice transplanted with a 1:1 mix of CD45.2 *Ccr2*^−/−^ and CD45.1 *Ccr2*^+/+^ BM cells, injected with 50 ng DT i.p. 4 weeks later and evaluated at day 7 after DT. **h**, Representative contour plot of Ly6C and CD64 expression within lung single live AM-excluded CD45^+^F4/80^+^SSC^lo^CD11b^+^ cells in CD45.1/CD45.2 IM^DTR^ mice treated with 50 ng DT i.p., transferred with CD45.1 BM wild-type cMo i.v. 24 h after DT and evaluated at days 2 and 14 after DT. Plots are representative of 5 mice, each of them giving similar results. Data show the mean ± s.e.m. and are pooled from two independent experiments (**c**, **e** and **g**: *n* = 4–8, 4–8 and 6 mice per group, respectively). *P* values were calculated by likelihood ratio tests (**b**), two-way ANOVA with Tukey’s post hoc tests (**c**,**e**) or one-way ANOVA with Sidak’s post hoc tests (**g**). *P < 0.05; **P < 0.01; ***P < 0.001; ****P < 0.0001. Source data

To assess whether the empty IM niche was repopulated by newly differentiated IMs, we performed flow cytometry time-course studies of lung myeloid cells after DT treatment in IM^DTR^ and control littermates. IM depletion occurred as early as 12 h after DT (Fig. 3a,b). IM numbers were still low in IM^DTR^ mice at day 2 and day 3 after DT compared to controls and this was associated with a significant increase in the numbers of lung cMo (Fig. 3a,b). From day 3 onwards, the numbers of IMs increased gradually to reach levels similar to the ones in control littermates at day 7 after DT (Fig. 3a,b). The influx of cMo into the lungs of DT-treated IM^DTR^ mice at day 2 after DT was preceded by a significant increase in the amount of the monocyte chemoattractant Ccl2 in the lung and serum 12 and 24 h after DT in IM^DTR^ mice compared to controls (Fig. 3c), suggesting that cMo were attracted to the lung in a Ccr2-dependent manner.

**Extended Data Fig. 4:**
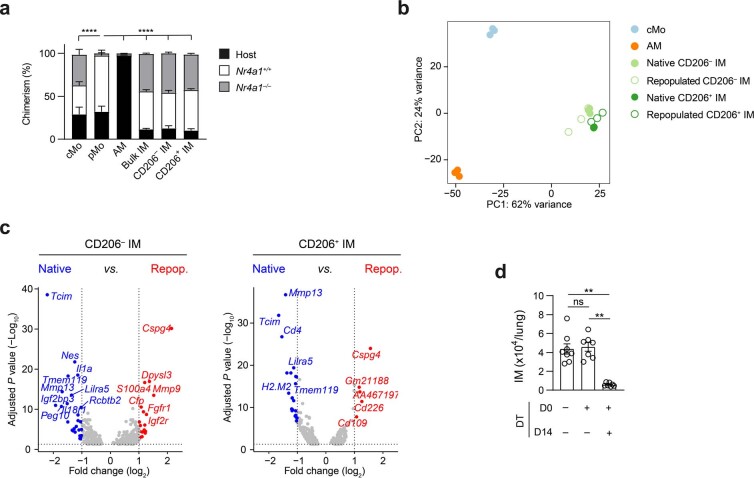
IM niche refilling is independent of Nr4a1 and repopulated IMs are largely similar to native IMs 14 days post-DT in IM^DTR^ mice. **a**, Bar graphs showing % of *Nr4a1*^*+/+*^ donor, *Nr4a1*^*−/−*^ donor and host chimerism in the indicated cell populations from lethally-irradiated, thorax-protected CD45.1/CD45.2 IM^DTR^ mice transplanted with a 1:1 mix of CD45.2 *Nr4a1*^*−/−*^ and CD45.1 *Nr4a1*^*+/+*^ BM cells, injected with 50 ng DT i.p. 4 weeks later and evaluated at day 7 post-DT. **b**, Principal Component (PC) analysis plot with % indicating the variability explained by each PC component, obtained by bulk RNA-seq analysis of lung cMo, AMs, CD206^−^ IMs and CD206^+^ IMs from untreated IM^DTR^ mice, and of lung CD206^−^ IMs and CD206^+^ IMs from DT-treated IM^DTR^ mice at day 14 post-DT (n = 3 pooled mice per replicate, 3 replicates per condition). **c**, Volcano plots depicting the DEG between native and repopulated CD206^−^ IMs (left) and native and repopulated CD206^+^ IMs (right). Transcripts significantly upregulated in native and repopulated IM subsets are colored in blue and red, respectively (log_2_ fold-change ± 1 and adjusted *P* value < 10^−2^). **d**, Bar graph showing lung IM numbers assessed by flow cytometry in IM^DTR^ mice treated or not with DT i.p. at day 0 and 14, and analyzed 24 h after the last DT treatment (day 15). Data show mean ± SEM and are pooled from 2 independent experiments (**a**,**d**) (a,d: *n* = 4,7-8 mice per group, respectively). *P* values were calculated using a one-way ANOVA with Tukey’s post-hoc tests, and compare donor CD45.1 *Nr4a1*^*+/+*^ chimerism between cell populations in **a**. Raw data and *P* values are provided as a source data file. ^**^, *P* < 0.01; ^****^, *P* < 0.0001. ns, not significant. Source data

To investigate whether cMo contributed to IM replenishment, we performed three sets of experiments. First, we generated chimeric mice in which lethally irradiated, thorax-protected CD45.2 IM^DTR^ mice were reconstituted with CD45.1 wild-type BM cells. At week 4 after transfer, the donor chimerism of blood cMo was 87%, while the donor chimerism of AMs and IMs was very low (Fig. 3d,e), indicating efficient BM donor reconstitution and thorax protection, respectively. When the chimeric IM^DTR^ mice were injected or not with DT at week 4 after transfer, the donor chimerism of IMs was significantly increased in the DT-treated chimeric IM^DTR^ mice (92%) compared to untreated counterparts at day 7 after DT, and reached levels similar to those observed in blood cMo (Fig. 3d,e), consistent with a major contribution of BM cells to the replenishment of the IM niche. Second, we generated BM competitive chimeras in thorax-protected CD45.1/CD45.2 IM^DTR^ mice engrafted with a 1:1 mix of CD45.1 *Ccr2*^+/+^ and CD45.2 *Ccr2*^−/−^ BM cells. At week 4 after reconstitution, only a few blood cMo were of donor *Ccr2*^*−/−*^ origin, as expected^26^ (Fig. 3f,g). When such competitive chimeras were injected or not with DT at week 4 after transfer, the majority of IMs (70%) were of donor *Ccr2*^+/+^ origin at day 7 after DT, comparable to the blood cMo (59%; Fig. 3f,g), indicating their dependency on Ccr2. Previous single-cell RNA-sequencing (scRNA-seq) analyses of lung CD64^+^ cells indicated, based on trajectory RNA velocity analyses, that Nr4a1-dependent CD16.2^+^ monocytes might represent precursors of CD206^−^ IMs^20^. BM competitive chimeras in thorax-protected CD45.1/CD45.2 IM^DTR^ mice engrafted with a 1:1 mix of CD45.1 *Nr4a1*^+/+^ and CD45.2 *Nr4a1*^−/−^ BM cells showed that donor *Nr4a1*^−/−^ chimerism of IMs (43%) was similar to donor *Nr4a1*^+/+^ chimerism of IMs (44%) at day 7 after DT (Extended Data Fig. 4a), indicating that IM replenishment was independent of Nr4a1 (ref. ^20^) and suggesting that CD16.2^+^ monocytes contributed minimally to IM repopulation as compared to Ccr2-dependent cMo. Third, we transferred CD45.1 wild-type BM Ly6C^+^ cMo intravenously (i.v.) into CD45.1/CD45.2 IM^DTR^ mice 1 d after DT. CD45.1^+^CD45.2^−^ cMo were mainly detected as Ly6C^+^CD64^−^ cells at day 2 after DT in the lung, while some CD45.1^+^CD45.2^−^ cells were detected as Ly6C^−^CD64^+^ cells at day 14 after DT in the lung (Fig. 3h), indicating that Ly6C^+^ cMo could differentiate into IM.

Finally, we analyzed lung cMo, AMs, CD206^+^ IMs and CD206^−^ IMs from untreated IM^DTR^ mice and repopulated lung CD206^+^ IMs and CD206^−^ IMs from DT-treated IM^DTR^ mice at day 14 after DT by bulk RNA-seq. Repopulated CD206^+^ IM and CD206^−^ IM subsets were largely similar to native IMs, with only 30 and 28 differentially expressed genes (DEGs) between native and repopulated CD206^+^ IMs and CD206^−^ IMs, respectively (log_2_ fold change ± 1, adjusted *P* value < 0.05; Extended Data Fig. 4b,c). Although *Tmem119* mRNA expression was lower in repopulated CD206^+^ IM and CD206^−^ IM subsets as compared to native CD206^+^ IM and CD206^−^ IM subsets, respectively (Extended Data Fig. 4c), they could still be efficiently re-depleted by DT at day 14 after first DT treatment (Extended Data Fig. 4d). Thus, similar to the steady-state situation^16,18,20^, Ccr2-dependent cMo could give rise to differentiated CD206^+^ IM and CD206^−^ IM subsets in DT-treated IM^DTR^ mice.

### scRNA-seq captures interstitial macrophage development from classical monocytes

**Fig. 4:**
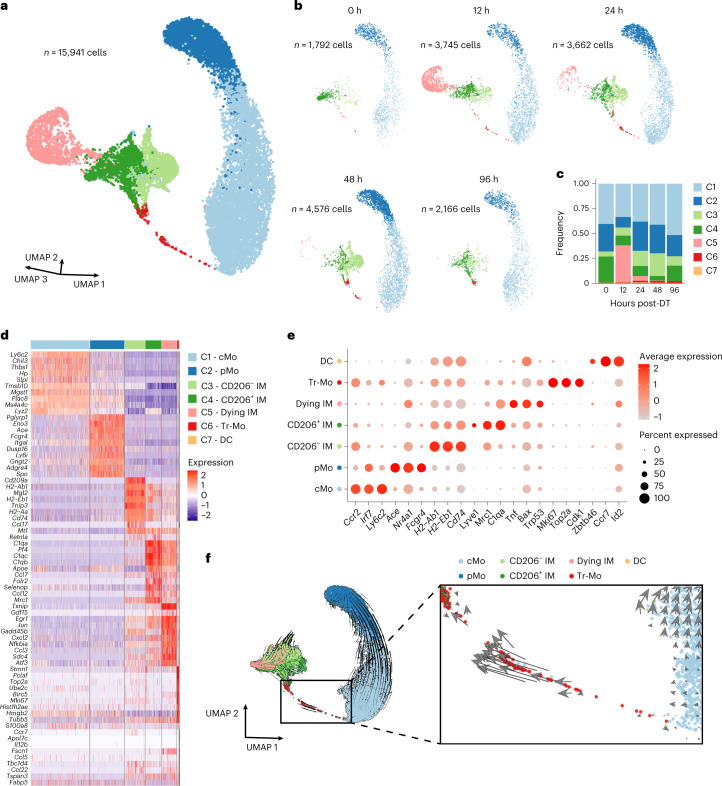
Time-course scRNA-seq analyses of interstitial macrophage niche refilling reveal discrete transitioning cells. **a**, Three-dimensional UMAP plot depicting the transcriptional identity of sorted lung CD45^+^SSC^lo^CD11b^+^F4/80^+^CD64^−^ monocytes and CD45^+^SSC^lo^CD11b^+^F4/80^+^CD64^+^ IMs merged from IM^DTR^ mice injected with DT i.p. at 0, 12, 24, 48 and 96 h before the analysis (*n* = 5 pooled mice per time point). **b**, UMAP plots from the five separate time points after DT, as in **a**. Inset indicates the number of cells analyzed (**a** and **b**). **c**, Histogram showing the frequency of each cluster at each time point after DT. **d**, Heat map depicting the single-cell expression of the ten most upregulated genes within each cluster. **e**, Dot plots show average expression of the indicated genes and the percentage of cells expressing the genes within each cluster. **f**, Prevalent pattern of RNA velocities substantiated by arrows and visualized on the same UMAP plot as shown in **a**. The square on the right shows a higher magnification of the area in the left square.

**Extended Data Fig. 5:**
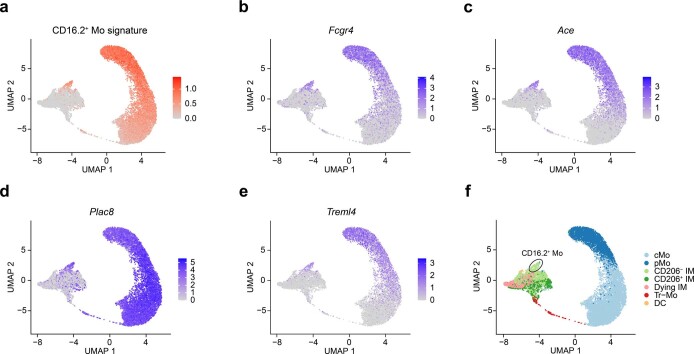
Identification of non-classical CD16.2^+^ monocytes in scRNA-seq data of monocyte-to-IM trajectory. UMAP feature plot depicting the transcriptional identity of sorted lung CD45^+^SSC^lo^CD11b^+^F4/80^+^CD64^−^ monocytes and CD45^+^SSC^lo^CD11b^+^F4/80^+^CD64^+^ IMs merged from IM^DTR^ mice injected with DT i.p. at 0, 12, 24, 48 and 96 hours before the analysis (n = 5 pooled mice per time point), according to their CD16.2^+^ monocyte (Mo) signature score (**a**) and to the expression of the indicated genes (**b-e**). **f**, Two-dimensional UMAP plot, as in **a**, identifying CD16.2^+^ monocytes.

Lung monocytes and IMs were sorted from five IM^DTR^ mice at 0, 12, 24, 48 and 96 h after DT and were subjected to single-cell droplet encapsulation with the 10x Genomics platform^27^, scRNA-seq and quality-control filtering. A total of 15,941 myeloid cells were analyzed and projected to global and time-specific uniform manifold approximation and projection (UMAP) plots (Fig. 4a,b), which led to the identification of seven distinct cell clusters (Fig. 4a–c). Based on differential expression analysis, we identified clusters corresponding to cMo (*Ccr2*, *Ly6c2*; cluster (C) 1), pMo (*Ace*, *Nr4a1*; C2), CD206^−^ IM (*H2-Ab1*, *Cd74*; C3) and CD206^+^ IM (*Lyve1*, *Mrc1*; C4; Fig. 4d,e). C3 not only encompassed CD206^−^ IM but also contained nonclassical CD16.2^+^ monocytes (*Fcgr4*, *Ace*)^20^ (Extended Data Fig. 5). C5 upregulated apoptosis-related genes (*Bax*, *Trp53*, *Tnf*), was almost uniquely present 12 h after DT and disappeared afterwards (Fig. 4b–e), likely representing DT-targeted native IM undergoing cell death, while C7 encompassed few contaminating DCs (*Zbtb46*, *Ccr7*; Fig. 4d,e). C6 encompassed cells expressing *Ccr2* and *Ly6c2* that were rare at steady state but enriched between 24 and 96 h after DT and made a transient bridge between cMo and a branching point leading to CD206^+^ IM and CD206^−^ IM subsets (Fig. 4b–e), which we named transitioning monocytes (Tr-Mo). RNA velocity analysis indicated that Tr-Mo moved from cMo toward IM subsets (Fig. 4f). These experiments thus captured the full pattern of monocyte-to-IM trajectory at the single-cell transcriptomic level.

### Transitioning monocytes transiently express cell cycling genes

**Fig. 5:**
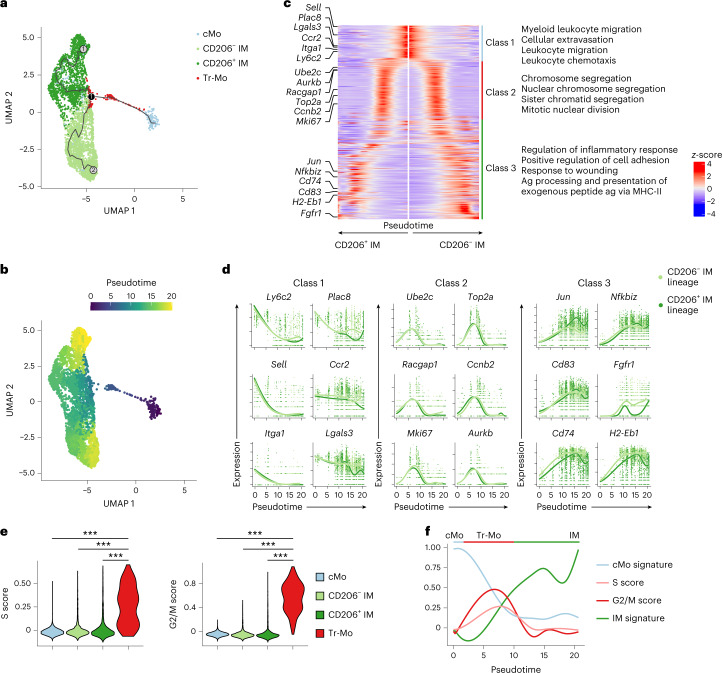
Trajectory analyses of interstitial macrophage development identify transient proliferating monocytes. **a**, Two-dimensional UMAP plot depicting the transcriptional identity and cell trajectories of lung cMo, Tr-Mo, CD206^−^ IMs and CD206^+^ IMs, as in Fig. 4a, evaluated by Monocle analysis. **b**, Two-dimensional UMAP plot depicting the pseudotime trajectory values of lung cMo, Tr-Mo, CD206^−^ IMs and CD206^+^ IMs, as in **a**. **c**, Heat map plot depicting the DEGs along pseudotime evaluated by tradeSeq in the common trajectory starting from cMo (middle) and ending in CD206^−^ IM and CD206^+^ IM subsets. DEGs are divided into three classes, and examples of genes and the main biological responses enriched in each class are represented on the left and right, respectively. **d**, Gene expression of the indicated genes along pseudotime evaluated by tradeSeq in both trajectories leading either to CD206^−^ IM or CD206^+^ IM subsets. **e**, S and G2/M cell cycle scores of single cells within cMo, Tr-Mo, CD206^−^ IMs and CD206^+^ IMs, as depicted by violin plots (height: score; width: abundance of cells). **f**, cMo and IM signatures, and S and G2/M scores depicted along pseudotime, as in **b**. *P* values were calculated by one-way ANOVA with Tukey’s post hoc tests (**e**). ^***^P < 0.001.

Next, we applied Monocle single-cell trajectory analysis^28^ to the scRNA-seq data encompassing cMo, Tr-Mo, CD206^+^ IMs and CD206^−^ IMs, and identified two main trajectories, both starting from cMo, moving across Tr-Mo until a branching point, and then bifurcating toward either CD206^−^ IMs or CD206^+^ IMs, in line with the real-time analysis (Fig. 5a,b). Genes that exhibited the same pattern of regulation along pseudotime in both CD206^+^ IM and CD206^−^ IM trajectories, as analyzed using tradeSeq^29^, encompassed three main classes of genes. First, cMo expressed genes enriched in cellular extravasation, leukocyte migration and chemotaxis (Fig. 5c,d), in line with tissue recruitment. Second, we observed a time-restricted upregulation of genes associated with cell proliferation (*Ube2c*, *Aurkb*, *Racgap1*, *Cdk1*, *Ccnb2*, *Mki67*; Fig. 5c,d) that peaked between 5 and 10 pseudotime units and corresponded to Tr-Mo, as attested by their elevated S and G2/M cell cycle score (Fig. 5e), indicative of DNA replication and mitosis, respectively. Such a state was then followed by increased expression of genes enriched in cell adhesion (Fig. 5c,d), supporting the idea of cell engraftment into their niche^16,20^. By mapping cMo signature, S and G2/M phases, as well as IM signature scores along pseudotime, we could sequentially observe the downregulation of cMo signature accompanied by an upregulation of cell division-related genes, which then decreased concomitantly to the acquisition of an IM signature that became predominant at the end of the trajectory (Fig. 5f). These data suggested that cMo, once in a vacant niche, became Tr-Mo that could reenter the cell cycle and expand before differentiating into CD206^+^ IM or CD206^−^ IM subsets.

### Transitioning monocytes proliferate locally in a Csf1r-dependent way

**Fig. 6:**
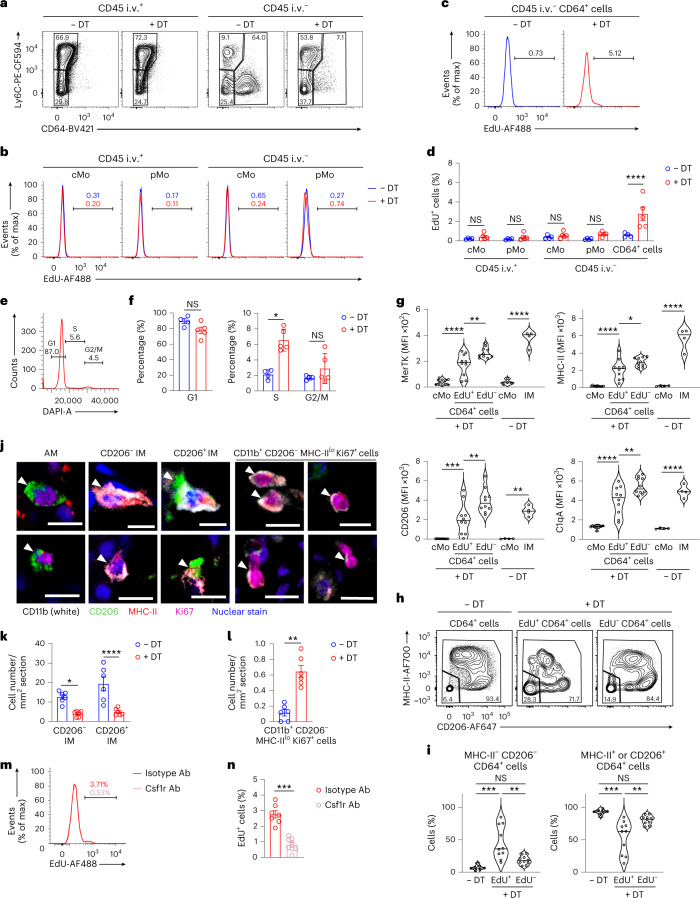
Transitioning monocytes can proliferate via Csf1r-dependent mechanisms. **a**, Representative plots of Ly6C and CD64 expression within lung CD45 i.v.^+^ and CD45 i.v.^−^ AM-excluded CD45^+^F4/80^+^SSC^lo^CD11b^+^ cells from EdU-pulsed IM^DTR^ mice treated or not with 50 ng DT i.p. 2 d before. **b**, Representative histograms of EdU levels in lung cMo and pMo, as in **a**. **c**, Representative histograms of EdU levels in lung CD64^+^ cells, as in **a**. **d**, Bar graphs showing the percentage of EdU^+^ cells in lung cMo and pMo, and in lung CD64^+^ cells, as in **a**. **e**, Representative histograms of DAPI signal in lung CD64^+^ cells, as in **a**. **f**, Bar graph showing the percentage of lung extravascular CD64^+^ cells in G1, S and G2/M phases, as in **e**. **g**, Expression of the indicated markers in lung cMo, EdU^+^CD64^+^ cells and EdU^−^CD64^+^ cells from EdU-pulsed IM^DTR^ mice at day 2 after DT and in lung cMo and IMs from untreated IM^DTR^ mice, as depicted by violin plots (height: MFI; width: abundance of cells). **h**, Representative plots of MHC-II and CD206 expression within lung CD64^+^ cells from untreated IM^DTR^ mice and EdU^−^/EdU^+^CD64^+^ cells from DT-treated EdU-pulsed IM^DTR^ mice, as in **g**. **i**, Percentage of MHC-II^−^ CD206^−^ cells and MHC-II^+^ or CD206^+^ cells within lung CD64^+^ cells from untreated IM^DTR^ mice and EdU^−^/EdU^+^ CD64^+^ cells from DT-treated EdU-pulsed IM^DTR^ mice, as in **h**, as depicted by violin plots (height: percentage; width: abundance of cells). **j**, Representative images of AMs, CD206^−^ IMs, CD206^+^ IMs and CD11b^+^CD206^−^MHC-II^lo^Ki67^hi^ cells, identified by confocal microscopy on lung sections from untreated and DT-treated IM^DTR^ mice, at day 2 after DT. **k**,**l**, Number of CD206^−^ IMs and CD206^+^ IMs (**k**) and CD11b^+^CD206^−^MHC-II^lo^Ki67^hi^ cells (**l**) per mm^2^, as in **j**. **m**, Representative histograms of EdU levels in lung CD64^+^ cells from DT-treated IM^DTR^ mice, as in **a**, and treated i.v. with Csf1r antibodies (Ab) or isotype control 6 and 28 h after DT. **n**, Bar graph showing the percentage of EdU^+^ cells in lung CD64^+^ cells, as in **m**. Data show the mean ± s.e.m. and are pooled from two independent experiments (**d**, **f**, **g**, **i**, **k**, **l** and **n**: *n* = 4–5, 4–5, 4–10, 7–10, 6, 6 and 7–8 mice per group, respectively). *P* values were calculated using a two-way ANOVA with Sidak’s post hoc tests (**b** and **k**), a two-tailed Mann–Whitney test (**e**, **l** and **n**) or one-way ANOVA with Tukey’s post hoc tests (**g** and **i**). ^*^P < 0.05; ^**^P < 0.01; ^***^P < 0.001; ^****^P < 0.0001. Scale bar, 10 µm. Source data

Next, we aimed to detect the Tr-Mo in vivo during the time of IM niche refilling. IM^DTR^ mice i.p. injected or not with DT were i.p injected with 5-ethynyl-2′-deoxyuridine (EdU) 4 h before the analysis, at day 2 after DT, a time point when the IM niche was depleted and Tr-Mo were detected by scRNA-seq. Mice were treated with CD45 antibodies i.v. 10 min before killing to discriminate blood from tissue cells by flow cytometry. Under these experimental conditions, virtually no EdU^+^ cells were detected in the intravascular and extravascular cMo (Fig. 6a–c), indicating that the EdU signal did not reflect a history of proliferation in the BM. Notably, we detected a significant increase in the percentage of EdU^+^ cells in the lung extravascular CD45^+^SSC^lo^CD11b^+^F4/80^+^CD64^int/hi^ cells (CD64^+^ cells hereafter; Fig. 6a), at day 2 after DT in IM^DTR^ mice as compared to non-treated IM^DTR^ mice (Fig. 6c,d). Moreover, staining with 4′,6-diamidino-2-phenylindole (DAPI) showed an increase in the percentage of CD64^+^ cells in the S phase at day 2 after DT in IM^DTR^ mice as compared to non-treated IM^DTR^ mice (Fig. 6e,f).

**Extended Data Fig. 6:**
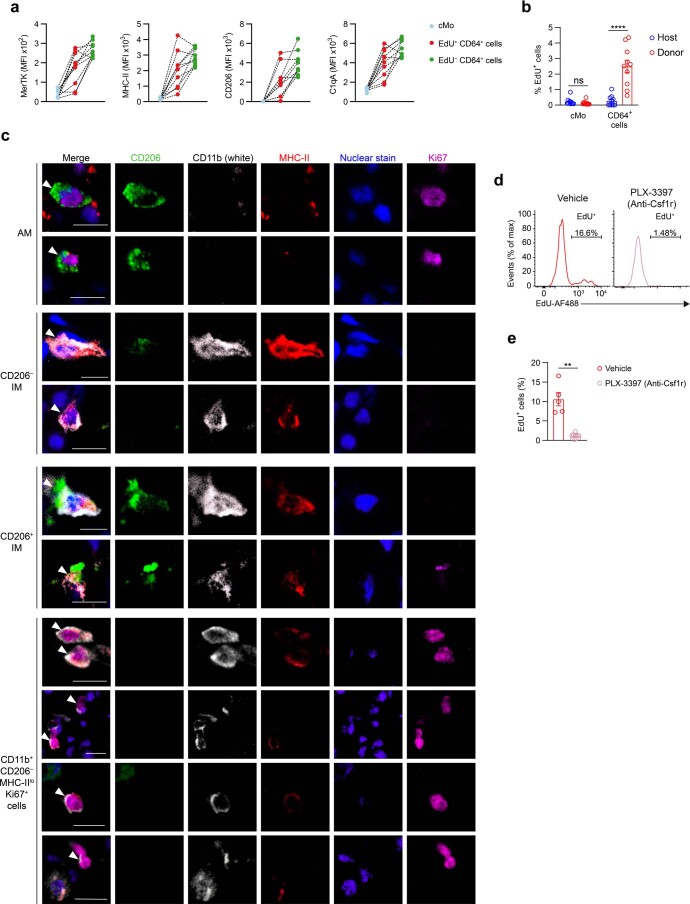
Tr-Mo are BM-derived cells in transition between cMo and IMs and whose proliferation is inhibited by Csf1r antagonists. **a**, Expression of the indicated markers in lung cMo, EdU^+^CD64^+^ and EdU^−^CD64^+^ cells from EdU-pulsed IM^DTR^ mice at day 2 post-DT. Values obtained from individual mice are connected with a dashed line. **b**, Bar graph showing the % of EdU^+^ cells in CD45.1 donor and CD45.2 host cells from lung cMo and CD64^+^ cells from lethally irradiated, thorax-protected CD45.2 IM^DTR^ mice reconstituted with CD45.1 BM wild-type cells, injected i.p. with DT and evaluated 2 days post-DT and 4 hours after EdU i.p. treatment by flow cytometry. **c**, Representative pictures of CD11b^−^CD206^hi^MHC-II^−^Ki67^+^ AMs, CD11b^+^CD206^lo^MHC-II^hi^ IMs (CD206^−^ IM), CD11b^+^CD206^hi^MHC-II^lo/int^ IMs (CD206^−^ IM) and CD11b^+^CD206^−^MHC-II^lo^Ki67^hi^ cells, identified by confocal microscopy on lung sections from untreated and DT-treated IM^DTR^ mice, at day 2 post-DT. **d**, Representative histograms of EdU levels in lung extravascular CD64^+^ cells from DT-treated IM^DTR^ mice that were treated with DT i.p. 3 days before, with the Csf1r antagonist PLX-3397 or with vehicle i.p. 1 and 2 days before, and with EdU i.p. 8 h before. **e**, Bar graph showing the % of EdU^+^ cells in lung extravascular CD64^+^ cells, as in **d**. Data are pooled from 2-3 independent experiments and show individual values (**a**) (*n* = 8 mice), or mean ± SEM (**b,e**) (b,e: *n* = 10,5-8 mice per group). *P* values were calculated using a two-way ANOVA with Tukey’s post hoc tests (**b**) or a two-tailed Student’s *t* test (**e**). Raw data and *P* values are provided as a source data file. ^**^, *P* < 0.01; ^****^, *P* < 0.0001; ns, not significant. Scale bar: 10 µm. Source data

Because RTMs can self-renew through proliferation, we tested whether the EdU^+^CD64^+^ cells corresponded to Tr-Mo or to differentiated IMs that were not depleted by DT treatment and underwent local proliferation. The expression of IM-associated markers such as MerTK, class II major histocompatibility complex (MHC-II), CD206 and C1qA^20^ on EdU^+^CD64^+^ cells was intermediate between that detected in cMo, which was significantly lower, and that detected in EdU^−^CD64^+^ cells, which was significantly higher than on EdU^+^CD64^+^ cells (Fig. 6g and Extended Data Fig. 6a), suggesting that EdU^+^CD64^+^ cells corresponded to the Tr-Mo transcriptional subset. While CD64^+^ cells from DT-untreated IM^DTR^ mice contained more than 90% of IMs (either as MHC-II^hi^CD206^int/lo^ IM or MHC-II^lo^CD206^+^ IM subsets^16,20^), the EdU^+^CD64^+^ cells from IM^DTR^ mice at day 2 after DT were significantly enriched in MHC-II^−^CD206^−^ monocytes (Fig. 6h,i). Conversely, like in DT-untreated IM^DTR^ mice, EdU^−^CD64^+^ cells from IM^DTR^ mice at day 2 after DT were mainly composed of MHC-II^hi^CD206^int/lo^ IM or MHC-II^lo^CD206^+^ IM subsets (Fig. 6h,i).

Next, we generated chimeric mice in which lethally irradiated, thorax-protected CD45.2 IM^DTR^ mice were reconstituted with CD45.1 wild-type BM donor cells. At day 2 after DT and 4 h after EdU i.p. injections, EdU^+^CD64^+^ cells were almost uniquely detected among donor cells, and not among host cells (Extended Data Fig. 6b). Confocal microscopy of lung sections from IM^DTR^ mice at day 2 after DT indicated efficient depletion of CD206^+^ IMs and CD206^−^ IMs and a significant increase in CD11b^+^CD206^−^MHC-II^lo^Ki67^hi^ monocytic proliferating cells compared to untreated IM^DTR^ mice (Fig. 6j–l and Extended Data Fig. 6c). Altogether, these data suggested that EdU^+^CD64^+^ cells represented BM-derived monocytes that proliferated locally and were in transition between cMo and differentiated IMs.

Csf1 receptor (Csf1r) signaling has an important role in the regulation of cell proliferation in the mononuclear phagocyte system^8,11,30–32^. To assess the contribution of Csf1r to the proliferation of EdU^+^CD64^+^ cells in our model, IM^DTR^ mice were injected with DT i.p. and treated i.v. with 250 µg mouse Csf1r antibodies or isotype control 6 and 28 h after DT. The percentage of EdU^+^ cells within CD64^+^ cells was significantly decreased in Csf1r antibody-treated mice as compared to isotype-treated DT-injected IM^DTR^ mice (Fig. 6m,n). We also treated DT-injected IM^DTR^ mice with the Csf1r small-molecule inhibitor pexidartinib (PLX3397) or vehicle i.p. at days 1 and 2 after DT and found that EdU incorporation was almost completely abrogated at day 3 after DT in CD64^+^ cells from PLX3397-treated mice compared to vehicle-treated counterparts (Extended Data Fig. 6d,e). In conclusion, EdU^+^CD64^+^ cells proliferated in the tissue through Csf1r-dependent mechanisms before differentiating into IMs.

### MafB restricts the proliferation and mediates interstitial macrophage development

**Fig. 7:**
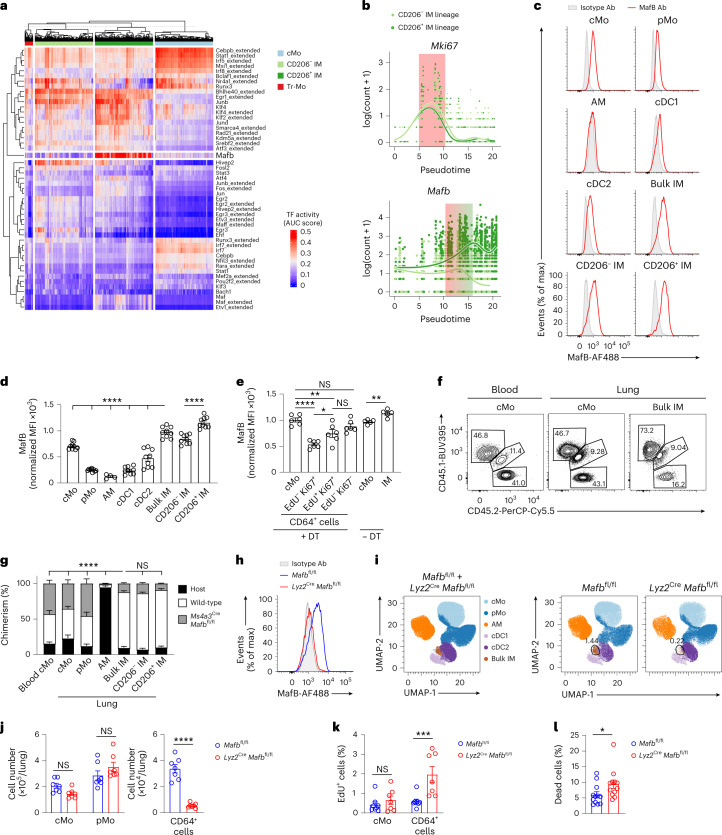
MafB restricts proliferation and mediates interstitial macrophage development. **a**, Heat map depicting predicted transcription factor (TF) activities across lung myeloid cells analyzed by scRNA-seq, as in Fig. 4a, as assessed by SCENIC. **b**, Expression of *Mki67* and *Mafb* along pseudotime evaluated by tradeSeq in both CD206^−^ IM or CD206^+^ IM trajectories, as in Fig. 5d. **c**,**d**, Representative histograms (**c**) and bar graphs showing normalized MFI (**d**) of MafB expression in the indicated lung myeloid cell populations from wild-type mice. **e**, Bar graphs showing expression of MafB in lung cMo and IMs from untreated IM^DTR^ mice, and in lung cMo, EdU^−^Ki67^+^, EdU^+^Ki67^+^ or EdU^−^Ki67^−^ CD64^+^ cells from EdU-pulsed IM^DTR^ mice at day 2 after DT. **f**,**g**, Representative CD45.1 and CD45.2 plots (**f**) and bar graphs showing the percentage of wild-type donor, *Ms4a3*^Cre^*Mafb*^fl/fl^ donor and host chimerism (**g**) in the indicated cell populations from lethally irradiated, thorax-protected CD45.1/CD45.2 IM^DTR^ mice transplanted with a 1:1 mix of CD45.2 *Ms4a3*^Cre^*Mafb*^fl/fl^ and CD45.1 wild-type BM cells, injected with 50 ng DT i.p. 4 weeks later and evaluated at day 7 after DT. **h**, Efficiency of *Mafb* depletion within lung CD64^+^ cells from *Lyz2*^Cre^*Mafb*^fl/fl^ mice evaluated by MafB intracellular staining. Data are representative of five mice, each of them giving similar results. **i**, Representative UMAP plots of lung CD45^+^CD11b^+^ or CD11c^+^ mononuclear cells analyzed by flow cytometry in *Lyz2*^Cre^*Mafb*^fl/fl^ and *Mafb*^fl/fl^ littermate controls (merged data from four mice per group). **j**–**l**, Absolute numbers of lung cMo, pMo and CD64^+^ cells (**j**), bar graphs showing the percentage of EdU^+^ cells within cMo and CD64^+^ cells (**k**) and bar graph showing the percentage of dead cells within CD64^+^ cells (**l**) from *Lyz2*^Cre^*Mafb*^fl/fl^ and *Mafb*^fl/fl^ mice. Data show the mean ± s.e.m. and are pooled from 2–3 independent experiments (**d**, **e**, **g**, **j**, **k** and **m**: *n* = 9, 5–6, 4–7, 7, 7–8 and 12 mice per group, respectively). *P* values were calculated using a one-way ANOVA with Tukey’s post hoc tests (**d**, **e** and **g**), a two-way ANOVA with Sidak’s post hoc tests (**j** and **k**) or a two-tailed Mann–Whitney test (**m**). In **d**, *P* values compare bulk IM versus every other population, or CD206^+^ IM versus CD206^−^ IM. In **g**, *P* values compare the percentage of donor CD45.1 wild-type chimerism. ^*^P < 0.05; ^**^P < 0.01; ^***^P < 0.001; ^****^P < 0.0001. AUC, area under the curve. Source data

To gain insights into the transcriptional control of the balance between Tr-Mo proliferation and IM differentiation, we applied the single-cell regulatory network inference and clustering (SCENIC) algorithm^33^ to our scRNA-seq data to map gene regulatory networks and predict transcription factor activities at the single-cell level. MafB was one of the high activity score transcription factors in CD206^+^ IMs (Fig. 7a). MafB restricts Csf1-dependent proliferation of myeloid progenitor cells in vivo^34^, as well as the self-renewal ability of macrophages^35,36^. TradeSeq trajectories showed that the transient upregulation of the cycling gene *Mki67* was followed by an increase in the expression of *Mafb* between 10 and 15 pseudotime units in both CD206^−^ and CD206^+^ IM trajectories (Fig. 7b), suggesting that MafB activation might restrict Tr-Mo proliferation and facilitate IM development from Tr-Mo. MafB intracellular staining of lung myeloid cells from wild-type C57BL/6 mice indicated an elevated expression of MafB in lung IMs, especially in CD206^+^ IMs, as compared to lung cMo, pMo, AMs and DCs (Fig. 7c,d). We also assessed the expression of MafB in lung CD64^+^ cells in EdU-pulsed IM^DTR^ mice at day 2 after DT. MafB expression was significantly lower in EdU^−^Ki67^+^ and EdU^+^Ki67^+^ CD64^+^ cells as compared to cMo (Fig. 7e), supporting that the proliferation of CD64^+^ cells required low expression of MafB.

**Extended Data Fig. 7:**
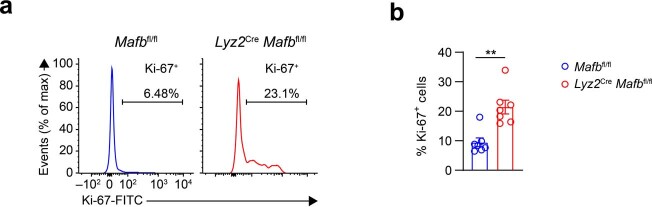
Lung CD64^+^ cells from *Lyz2*^Cre^*Mafb*^fl/fl^ mice exhibit a higher proliferative potential as compared to those from littermate controls. **a**, Representative histograms of Ki67 stainings within lung lung extravascular CD45^+^SSC^lo^CD11b^+^F4/80^+^CD64 ^int/hi^ cells (CD64^+^ cells) from *Lyz2*^Cre^*Mafb*^fl/fl^ and littermate controls. Insets indicate the % of Ki67^+^ cells within CD64^+^ cells. **b**, Bar graph showing the % of Ki67^+^ cells within lung CD64^+^ cells. Data show mean ± SEM and are pooled from 2 independent experiments (**b**) (*n* = 7 mice per group). *P* values were calculated using an unpaired two-tailed Mann-Whitney test. Raw data and *P* values are provided as a source data file. ^***^, *P* < 0.001. Source data

Next, we generated C57BL/6 *Mafb*^fl/fl^ mice and crossed them with mice constitutively expressing Cre recombinase under the control of the lysozyme M promoter (*Lyz2*^Cre^) or the Ms4a3 promoter (*Ms4a3*^Cre^) to generate mice with myeloid-restricted *Mafb* deficiency. To assess whether MafB mediated IM development from cMo in vivo, we generated BM competitive chimeras in thorax-protected CD45.1/CD45.2 IM^DTR^ mice engrafted with a 1:1 BM cell mix from CD45.1 wild-type and CD45.2 *Ms4a3*^Cre^*Mafb*^fl/fl^ mice. At day 7 after DT, evaluation of myeloid cell chimerism in the lung indicated that myeloid-restricted *Mafb* deficiency strongly impaired the ability of cMo to repopulate the niches of both IM subsets (Fig. 7f,g). MafB protein was absent in CD64^+^ cells from *Lyz2*^Cre^*Mafb*^fl/fl^ mice (Fig. 7h) and the numbers of CD64^+^ cells were significantly decreased in *Lyz2*^Cre^*Mafb*^fl/fl^ mice compared to *Mafb*^fl/fl^ mice, while the numbers of cMo and pMo were identical (Fig. 7i,j). Ki67 staining indicated an increased proliferative ability of the few CD64^+^ cells present in *Lyz2*^Cre^*Mafb*^fl/fl^ mice compared to *Mafb*^fl/fl^ mice (Extended Data Fig. 7). Similarly, i.p. EdU treatment 4 h before killing indicated a significant increase in the percentage of EdU^+^ cells within CD64^+^ cells from *Lyz2*^Cre^*Mafb*^fl/fl^ mice compared to littermate controls (Fig. 7k). Finally, we found that the percentage of dead cells was significantly higher in CD64^+^ cells from *Lyz2*^Cre^*Mafb*^fl/fl^ mice compared to littermate controls (Fig. 7l), suggesting that the higher proliferative rate observed in MafB-deficient CD64^+^ cells did not lead to an increase in the number of CD64^+^ cells because of the increased cell death. Altogether, these data suggested that MafB could restrict the proliferation of lung CD64^+^ cells and mediate IM development.

### MafB and c-Maf differentially control lung interstitial macrophage identity

**Extended Data Fig. 8:**
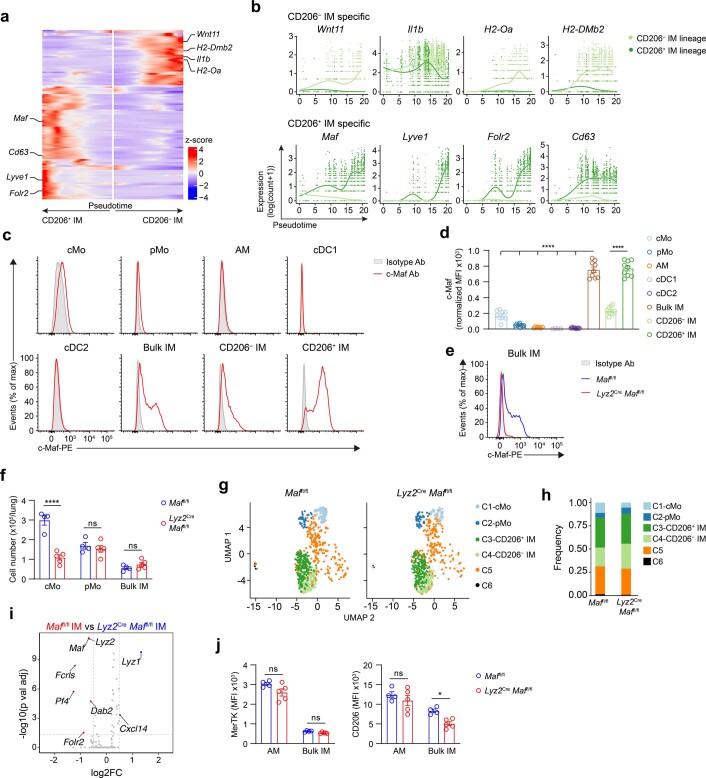
C-Maf specifically controls the identity of the CD206^+^ IM subset. **a**, Heatmap plot depicting the DEG along pseudotime evaluated by tradeSeq in the subset-specific trajectories starting from cMo (middle) and ending in CD206^−^ IM and CD206^+^ IM subsets. **b**, Gene expression of the indicated genes along pseudotime evaluated by tradeSeq in both trajectories leading either to CD206^−^ IM or CD206^+^ IM subsets. **c**, Representative histograms of c-Maf protein expression in the indicated lung myeloid cell populations from wild-type mice. **d**, Bar graphs showing normalized MFI of c-Maf expression, as in **c**. *P* values compare bulk IMs vs. every other population, or CD206^+^ IMs vs. CD206^−^ IMs. **e**, Efficiency of *Maf* depletion within lung IMs from *Lyz2*^Cre^*Maf*^fl/fl^ mice evaluated by c-Maf protein intracellular staining. Data are representative of 5 mice, each of them giving similar results. **f**, Absolute numbers of lung cMo, pMo and IMs quantified by flow cytometry in *Lyz2*^Cre^*Maf*^fl/fl^ and *Maf*^fl/fl^ mice. **g**, UMAP plot depicting the transcriptional identity of lung CD45^+^SSC^lo^CD11b^+^F4/80^+^ CD64^−^ monocytes and CD64^+^ cells from *Lyz2*^Cre^*Maf*^fl/fl^ mice and littermate controls (n = 5 pooled mice per group). **h**, Histogram showing frequency of each cluster in *Lyz2*^Cre^*Maf*^fl/fl^ and *Maf*^fl/fl^ mice. **i**, Volcano plot depicting the DEG between lung IMs (C3) from *Lyz2*^Cre^*Maf*^fl/fl^ and *Maf*^fl/fl^ mice. Transcripts significantly upregulated in IMs from *Maf*^fl/fl^ and *Lyz2*^Cre^*Maf*^fl/fl^ mice are colored in red and blue, respectively (log_2_ fold-change ± 0.5 and adjusted *P* value < 0.05). **j**, Surface expression of MerTK and CD206 in lung AMs and IMs, quantified by flow cytometry in *Lyz2*^Cre^*Maf*^fl/fl^ and *Maf*^fl/fl^ mice. (**d,f,j**) Data show mean ± SEM and are pooled from 3 independent experiments (*n* = 9 mice per group) (**d**) or 2 independent experiments (n = 4-5 mice per group) (**f,j**). *P* values were calculated using a one-way ANOVA with Tukey’s post-hoc test (for bulk IMs) or a two-tailed Mann-Whitney test (for IM subsets), or a two- way ANOVA with Tukey’s post-hoc test (**f,j**). Raw data and *P* values are provided as a source data file. ^*^, *P* < 0.05; ^****^, *P* < 0.0001; ns, not significant. Ab, antibody. Source data

Next, we investigated to what extent the identity of CD64^+^ cells was impacted by myeloid-restricted MafB deficiency, as well as the contribution of myeloid-restricted c-Maf to IM maintenance and identity. MafB and c-Maf are b-ZIP transcription factors that belong to the same family of large Maf proteins^37^ and can cooperate together in some contexts, such as in the regulation of macrophage self-renewal^35,38^. Maf activity (Fig. 7a), *Maf* gene expression (Extended Data Fig. 8a,b) and Maf protein expression (Extended Data Fig. 8c,d) were elevated in CD206^+^ IMs compared to CD206^−^ IMs. Nevertheless, unlike in *Lyz2*^Cre^*Mafb*^fl/fl^ mice, IM numbers were normal in *Lyz2*^Cre^*Maf*^fl/fl^ mice (Extended Data Fig. 8e,f).

**Fig. 8:**
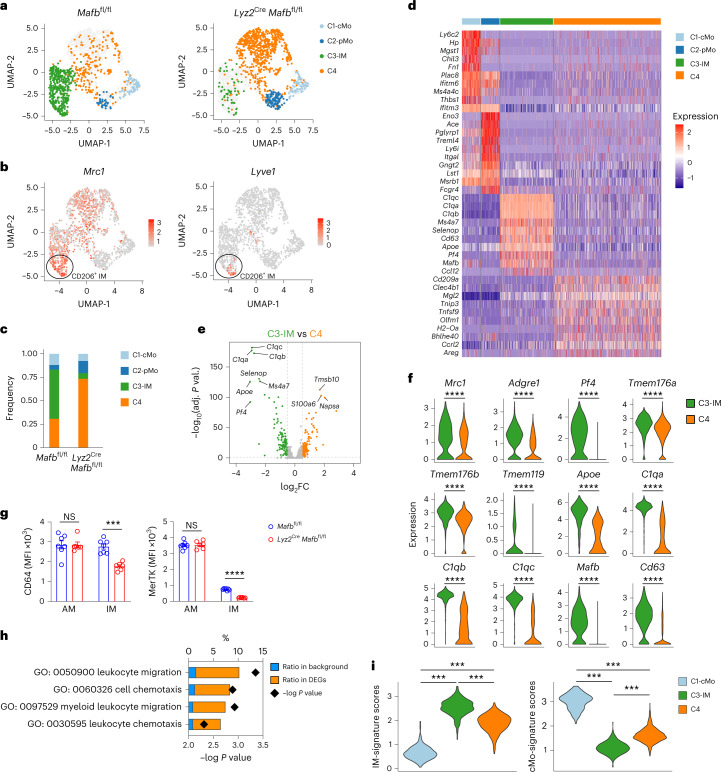
Interstitial macrophage identity is severely impaired in myeloid-restricted *Mafb*-deficient mice. **a**, UMAP plots depicting the transcriptional identity of lung CD45^+^SSC^lo^CD11b^+^F4/80^+^ CD64^−^ monocytes and CD64^+^ cells from *Lyz2*^Cre^*Mafb*^fl/fl^ mice and littermate controls (*n* = 5 pooled mice per group). **b**, UMAP feature plots representing single-cell expression of *Mrc1* and *Lyve1* in lung myeloid cells merged from *Lyz2*^Cre^*Mafb*^fl/fl^ mice and littermate controls, as in **a**. **c**, Histogram showing frequency of each cluster in *Lyz2*^Cre^*Mafb*^fl/fl^ mice and littermate controls. **d**, Heat map depicting the single-cell expression of the ten most upregulated genes within each cluster. **e**, Volcano plot depicting DEGs between C3 and C4 clusters. Transcripts significantly upregulated in C3 and C4 are colored in green and orange, respectively (log_2_ fold change ± 0.5 and adjusted *P* value < 0.05). **f**, Expression of the indicated genes within C3 and C4 clusters, as depicted by violin plots (height: expression; width: abundance of cells). **g**, Surface expression of CD64 and MerTK in lung AMs and CD64^+^ cells, evaluated by flow cytometry in *Lyz2*^Cre^*Mafb*^fl/fl^ and littermate controls. **h**, GO enrichment analysis performed on the upregulated genes in C4 as compared to C3. **i**, IM and cMo signature scores within C1, C3 and C4 clusters, as depicted by violin plots (height: scores; width: abundance of cells). Data show the mean ± s.e.m. and are pooled from two independent experiments (**g**; *n* = 6–7 mice per group). *P* values were calculated using a Wilcoxon rank sum test (**e** and **f**), a two-way ANOVA with Sidak’s post hoc test (**g**), a two-tailed Mann–Whitney *U* test with Benjamini–Hochberg false discovery rate correction (**h**), or a one-way ANOVA with Tukey’s post hoc test (**i**). ^***^P < 0.001; ^****^P < 0.0001. Source data

Hence, we performed scRNA-seq analysis of lung cMo, pMo and CD64^+^ cells from *Lyz2*^Cre^*Mafb*^fl/fl^, *Lyz2*^Cre^*Maf*^fl/fl^ and control littermates. Compared to the lungs of *Mafb*^fl/fl^ or *Maf*^fl/fl^ control mice, which contained cMo (C1), pMo (C2) and CD206^+^ IMs and CD206^−^ IMs (C3), *Lyz2*^Cre^*Mafb*^fl/fl^ mice lacked all IMs in the lung (C3), while a transcriptionally distinct cluster of cells (*Clec4b1*, *Mgl2*, *Tnip3*; C4) was enriched instead (Fig. 8a–d), suggesting that the few CD64^+^ cells present in *Lyz2*^Cre^*Mafb*^fl/fl^ mice had a completely different transcriptional profile than wild-type IMs. Of note, we found 216 DEGs (log_2_ fold change ± 0.5, adjusted *P* value < 5 × 10^−2^) between the wild-type IM cluster (C3) and the cluster enriched in *Lyz2*^Cre^*Mafb*^fl/fl^ mice (C4; Fig. 8e). The expression of prototypical IM identity genes (*Mrc1*, *Adgre1*, *Pf4*, *Tmem176a*, *Tmem176b*, *Tmem119*, *Apoe*, *C1q*, *Mafb*, *Cd63*)^20^ was significantly decreased in C4 as compared to wild-type IMs (C3; Fig. 8f). Flow cytometry of CD64^+^ cells from *Lyz2*^Cre^*Mafb*^fl/fl^ mice indicated that they exhibited decreased expression of CD64 and MertK protein compared to those from *Mafb*^fl/fl^ control littermates (Fig. 8g). In addition, the profile of C4 in *Lyz2*^Cre^*Mafb*^fl/fl^ mice was enriched in biological responses similar to those found at the beginning of the cMo-to-IM trajectory, such as leukocyte migration and chemotaxis (Fig. 8h) and was intermediate between cMo and IMs (Fig. 8i). Conversely, we found only a few DEGs between IMs from *Maf*^fl/fl^ and *Lyz2*^Cre^*Maf*^fl/fl^ mice (Extended Data Fig. 8g–i). *Folr2* was among the genes significantly downregulated in c-Maf-deficient IMs compared to wild-type IMs (Extended Data Fig. 8i), suggesting that the identity of the CD206^+^ IM subset was regulated by c-Maf^20^. In line with this, CD206 protein expression was significantly decreased in IMs from *Lyz2*^Cre^*Maf*^fl/fl^ mice compared to those from *Maf*^fl/fl^ controls (Extended Data Fig. 8j), suggesting that c-Maf had a specific role in the CD206^+^ IM subset. Altogether, these data showed that CD64^+^ cells from *Lyz2*^Cre^*Mafb*^fl/fl^ mice shared similarities with Tr-Mo, and indicated a severe impairment of IM development and identity in the absence of MafB.

## Discussion

Here, we found that tissue cMo that transitioned toward IMs had the ability to proliferate locally in a vacant niche, in a MafB-restricted fashion, before undergoing differentiation into distinct IM subsets. We used a model of lung IM niche depletion and refilling that allowed us to characterize the transient, Csf1r-dependent, proliferation of monocytes, which would be difficult to capture in a steady-state setting. These observations shed new light on the complex regulation of monocyte proliferation versus RTM differentiation.

In the IM^DTR^ model, we defined IMs by the combined expression of *Cx3cr1* and *Tmem119*, a marker that was previously considered to be microglia specific^39^. Around 50 ng DT specifically depleted lung IMs in IM^DTR^ mice, while microglia were depleted only at a higher dose. This might be due to lower access of DT to the brain, due to the blood–brain barrier, or a lower sensitivity of microglia to DT-induced cell death, or both. The efficient and specific IM depletion observed in DT-treated IM^DTR^ mice suggested that sustained Cre expression under the control of *Tmem119* and high expression of *Cx3cr1*, as observed in IMs, are both required to render cells sensitive to DT-induced cell death.

In adult mice, IMs are maintained by BM-derived Ccr2-dependent cMo at steady state^16,18,20,40^. Here, we showed that the IM^DTR^ mice accurately recapitulated the steady-state ontogeny of IMs, albeit in an accelerated way, and as such represented a relevant tool to study monocyte-to-IM trajectories. Nr4a1-dependent CD16.2^+^ monocytes have been suggested to be putative precursors of CD206^−^ IMs^20^, but this was uniquely based on scRNA-seq trajectory analyses. While a contribution of CD16.2^+^ monocytes to CD206^−^ IMs cannot be ruled out, no definitive proof exists so far, and their contribution would arguably be minimal as compared to the one of Ccr2-dependent cMo.

The current view is that, in the myeloid compartment, the ability to proliferate is limited to progenitor cells and mature RTMs. RTM maintenance is thus thought to be achieved through either the self-renewal of differentiated RTM, or the recruitment of monocytes that differentiate into RTMs in a tissue-specific manner^4–6,8,11,15,30,38^. Our results showed that monocytes can also proliferate in vacant tissue niches to contribute to RTM development in vivo. Such monocytes arose from cMo and could proliferate transiently in the tissue through Csf1r-dependent mechanisms. Our data are consistent with the idea that a limited number of cMo can give rise to a larger number of RTMs in tissues through a sequence of events involving first a proliferation in response to local Csf1r ligands, followed by the activation of common and subset-specific transcriptional programs that drive RTM differentiation. While the relative contribution of the Csf1r ligands Csf1 and IL-34 to monocyte proliferation remains to be determined, reports indicating that IM maintenance requires Csf1 rather than IL-34 would be consistent with a preferential contribution of the Csf1–Csf1r axis to this process^32,41,42^.

Tr-Mo proliferation occurred before the branching toward CD206^−^ IMs or CD206^+^ IMs and the phenotype of EdU^+^CD64^+^ cells was intermediate between those of cMo and IMs. We found that the majority of EdU^+^CD64^+^ cells did not express the IM markers MHC-II or CD206 and were bona fide tissue monocytes. The high variability observed in the expression levels of IM markers in EdU^+^CD64^+^ cells further suggested that this cell state was highly dynamic.

*Mafb* expression slightly increased in CD206^−^ IM and CD206^+^ IM trajectories after the upregulation of cycling genes, and MafB expression was decreased in proliferating monocytes and increased in IMs compared to cMo. MafB restricts Csf1-dependent proliferation in myeloid progenitor cells^34,43^, as well as the self-renewal ability of differentiated macrophages^35^, linking MafB activity with Csf1 responsiveness and the balance between proliferation and differentiation. Our data are consistent with the hypothesis that low MafB expression is required for monocyte proliferation, while subsequent increased MafB expression would restrict proliferation and drive their differentiation into IMs. Supporting this claim, *Mafb*-deficient CD64^+^ cells exhibited an increased proliferation potential compared to wild-type IMs and seemed to be held in a pre-macrophage state. Our results emphasized a differential requirement for MafB and c-Maf in lung IM development, as c-Maf deficiency was uniquely associated with changes that were restricted to CD206^+^ IMs, which have been shown to be preferentially associated with the vasculature^16^. Of note, c-Maf was reported to regulate perivascular RTM phenotypes across different tissues^44^.

RTM depletion is commonly observed in various inflammatory contexts, and this creates vacant niches that need to be refilled^12^. Whether local monocyte proliferation occurs under other homeostatic or natural challenge situations associated with RTM depletion or expansion remains to be investigated. Our findings support the idea that systemic blood measurements of monocyte responses might not appropriately reflect the actual immune responses and immunopathology occurring in peripheral tissues. Further understanding the molecular basis underlying Csf1r-dependent monocyte proliferation in peripheral organs will be crucial to manipulate such pathways for preventive or therapeutic purposes.

## Methods

### Mice

The following mice on the C57BL/6 background were used in this study: CD45.2 wild-type C57BL/6 (The Jackson Laboratory), CD45.1 wild-type C57BL/6J (The Jackson Laboratory, 002014), *Cx3cr1*^GFP/+^ (ref. ^45^; The Jackson Laboratory, 005582), *Tmem119*^Cre^ (see below), *Rosa26*^LSL-EYFP^ (ref. ^23^; The Jackson Laboratory, 006148), *Cx3cr1*^LSL-DTR/+^ (ref. ^25^; The Jackson Laboratory, 025629), *Ccr2*^*−/−*^ (ref.^46^; The Jackson Laboratory, 004999), *Nr4a1*^*−/−*^(ref.^47^ ;The Jackson Laboratory, 006187), *Maf*^fl/fl^ (ref. ^48^; kindly provided by F. Andris), *Mafb*^fl/fl^ (generated by D.P. and the GIGA Mouse facility and Transgenics Platform, Liège University, Belgium, see below), *Lyz2*^Cre^ (ref. ^49^; The Jackson Laboratory, 004781) and *Ms4a3*^Cre^ (ref. ^9^). Myeloid-restriced *Maf* or *Mafb* depletion was achieved by crossing *Maf*^fl/fl^ or *Mafb*^fl/fl^ mice with *Lyz2*^Cre^ or *Ms4a3*^Cre^ mice.

C57BL/6 *Tmem119*^Cre^ knock-in mice were generated using CRISPR/Cas-mediated genome engineering by Cyagen Bioscience. In brief, the *Tmem119* targeting vector was designed by cloning a genomic fragment encompassing exon 2 of the *Tmem119* gene from BAC clones RP23-187D5 and RP23-126P3. A *Cre-polyA* cassette was introduced in the *Tmem119* targeting vector upstream of the ATG start codon between a 2.1-kb 5′ homology arm and a 2.1-kb 3′ homology arm. *Tmem119*-gRNA (protospacer, CAGGGGACCATGTTGAGCTATGG), *Cas9* mRNA and *Tmem119* targeting vector were co-injected into pronuclei of C57BL/6J one-cell-stage zygotes, followed by implantation of the zygotes into surrogate mothers to obtain targeted knock-in offspring. F0 knock-in founder animals were identified by PCR followed by sequence analysis. *Tmem119*^Cre/+^ mice were then back-crossed to CD45.2 or CD45.1 C57BL/6J mice for at least four generations. *Tmem119*^Cre^ mice were genotyped by PCR using the following primers: PCR primers 1 for mutant allele (annealing temperature, 60.0 °C): forward primer: 5′- TCCGTAACCTGGATAGTGAAACAG-3′; reverse primer: 5′-ATATGTCCTTCCGAGTGAGAGAC-3′; product size: 270 bp (mutant). PCR primers 2 for wild-type allele (annealing temperature, 60.0 °C): forward primer: 5′-ACCGAGGACAGAAATGAATAAGATG-3′; reverse primer: 5′-AGGGAACGAGGATGGGTAGTAG-3′; product size: 643 bp (wild type).

C57BL/6 *Mafb*^fl/fl^ mice were generated using recombination-mediated genetic engineering. Briefly, the genomic segment covering the *Mafb* single exon was retrieved to PL253 vector using BAC recombineering. The loxP-EM7-Neo-loxP cassette was cloned by PCR from PL452 plasmid and ligated to the Mafb 5′ segment (PL253/Mafb/Neo 5′) and the cassette was ‘popped out’ by electroporating to SW106 cells expressing Cre and 5′ loxP left in the construct. The FRT-Neo-FRT-loxP cassette was cloned from PL451 plasmid and ligated to the Mafb 3′ segment. The purified plasmid was electroporated into mouse embryonic stem cells and the cells were selected under G418 treatment for 1 week. The bona fide clones with successful homologous recombination were screened by Southern blot. Successfully recombined clones were injected into blastocysts to make *Mafb*^fl^-Neo mice. These mice were crossed to an *FLP*-expressing line to remove the Pgk-Neo cassette and generate *Mafb*^fl^ mice. *Mafb*^fl^ mice were genotyped by PCR using the following primers: forward primer: 5′- TCCATCCATCTTGGGAAAAG-3′; reverse primer: 5′-TCAGGACTGGGCTGCTAGTT-3′; product size: 320 bp (Mutant), 220 bp (wild type).

*Tmem119*^Cre^ and *Rosa26*^LSL-EYFP^ mice were crossed to create *Tmem119*^Cre^*Rosa26*^LSL-EYFP^ mice. *Tmem119*^Cre^ and *Cx3cr1*^LSL-DTR/+^ mice were crossed to create *Tmem119*^Cre^*Cx3cr1*^LSL-DTR/+^ mice, referred to as ‘IM^DTR^’ mice. Since we observed some YFP labeling in CD45^−^ cells in the testis and ovaries of *Tmem119*^Cre^*Rosa26*^LSL-EYFP^ mice, we did not use *Tmem119*^Cre^*Rosa26*^LSL-EYFP^ or *Tmem119*^Cre^*Cx3cr1*^LSL-DTR^ mice as breeders to avoid any issues arising from germline recombination. CD45.1/CD45.2 IM^DTR^ mice were generated by crossing CD45.1 *Tmem119*^Cre^ with CD45.2 *Cx3cr1*^LSL-DTR^ mice.

No sex-specific differences were observed in pilot experiments. A mix of male and female mice between 6 and 10 weeks of age were used for each experiment, except for chimera experiments where mice between 11 and 15 weeks of age were used. The mice were bred and housed under specific-pathogen-free conditions at the GIGA Institute (Liège University, Belgium), maintained in a 12-h light-dark cycle, and had access to normal diet chow and water ad libitum. Mice were identified according to genotype and all experiments were performed with age-matched and sex-matched littermates. For Csf1r-blocking experiments, mice were randomly assigned to vehicle or isotype antibodies and anti-Csf1r treatments. For experiments using IM^DTR^ mice that were treated or not with DT, mice were randomly allocated to DT treatment or not. Investigators were not blinded during the collection and analysis of the data, except for the quantification of microscopy lung sections, where investigators were blinded.

All animal experiments described in this study were reviewed and approved by the Institutional Animal Care and Use Committee of the University of Liège (ethical approval no. DE1956). The ‘Guide for the Care and Use of Laboratory Animals,’ prepared by the Institute of Laboratory Animal Resources, National Research Council, and published by the National Academy Press, as well as European and local legislations, was followed carefully. Accordingly, the temperature and relative humidity were 21 °C and 45–60%, respectively.

### Reagents and antibodies

A complete list of the reagents, antibodies and commercial assays used in this paper can be found in Supplementary Table 1.

### In vivo treatments with chemicals and antibodies

For DT-induced depletion of IM, IM^DTR^ mice were injected i.p. with a single dose of 50 ng DT (List Biological Labs, 150), unless otherwise stated. Control mice were either untreated IM^DTR^ mice, or *Tmem119*^Cre/+^ littermate control mice injected with DT. For EdU incorporation experiments, IM^DTR^ mice were injected i.p. with 1 mg EdU (Santa Cruz Biotechnology, sc-284628) in 200 µl PBS 4 h before killing, unless otherwise stated. For all experiments involving EdU incorporation, 1 µg of PerCP-Cy5.5-conjugated anti-mouse CD45 (clone 104, BD Biosciences, 552950) was i.v. injected 10 min before killing to distinguish blood circulating (CD45-PerCP-Cy5.5^+^) and tissue leukocytes (CD45-PerCP-Cy5.5^−^). For Csf1r-blocking experiments, 250 µg of anti-mouse Csf1r-blocking antibody (clone AFS98, Bio X Cell, BE0213) or isotype control (clone 2A3, Bio X Cell, BE0089) was injected i.v. 6 and 28 h after DT injection. For experiments with Csf1r inhibitors, 100 mg per kg body weight of pexidartinib (PLX3397; MedChemExpress, HY-16749) was injected i.p. 24 and 48 h after DT injection.

### Bone marrow, blood and tissue leukocyte isolation

Blood was collected by retro-orbital plexus bleeding of terminally anesthetized mice. Mice were then euthanized by cervical dislocation. Peritoneal lavage was obtained by injecting 10 ml HBSS (Lonza, BE10-508F) into the peritoneal cavity and collecting the washout. Mice were then perfused with 10 ml PBS via the left ventricle, and lungs, brain, liver, spleen, intestine and colon were dissected.

For BM cells, femurs and tibias were dissected and cleaned of soft adhering tissue. Distal and proximal ends were opened, and BM cells were flushed out. After centrifugation, cell pellets were resuspended in ice-cold PBS (Thermo Fisher, 14190094) containing 10 mM EDTA (Merck Millipore, 1084181000) and cell suspensions were filtered using a cell strainer (70 µm, Corning, 352350) to obtain a single-cell suspension.

Lungs, brains, liver and spleen were cut into small pieces with razor blades, and digested for 1 h at 37 °C in HBSS containing 5% vol/vol FBS (Thermo Fisher, 10270098), 1 mg ml^−1^ collagenase A (Sigma, 14190094) and 0.05 mg ml^−1^ DNase I (Sigma, 11284932001). After 45 min of digestion, the suspension was flushed using a 18-gauge needle to dissociate aggregates. Ice-cold PBS (Thermo Fisher, 14190094) containing 10 mM EDTA (Merck Millipore, 1084181000) was added to stop the digestion process and cell suspensions were filtered using a cell strainer (70 µm, Corning, 352350). Mononuclear leukocytes from lungs and livers were enriched using a Percoll density gradient (GE Healthcare, 17089101) and by harvesting cells from the 1.080:1.038 g ml^−1^ interface.

For the isolation of leukocytes from the small intestines and colons, small intestines and colons were dissected from the pylorus and the rectum, were separated from the mesenteric tissue from Peyer’s patches and from fat and were placed in ice-cold HBSS with 2% FBS. Intestinal content was removed with PBS, and the small intestines and colons were opened by a longitudinal cut and washed three times in ice-cold HBSS with 2% FBS. To remove mucus and epithelial cells, small intestines and colons were incubated with HBSS with 2% FBS and 1 mM 1,4 dithiothreitol (Sigma, 10197777001) for 20 min with constant shaking followed by an incubation with HBSS containing 2% FBS and 1.3 mM EDTA for 40 min. Tissue pieces were then cut into small pieces and incubated for 1 h at 37 °C with RPMI containing 2% FBS, 2 mg ml^−1^ collagenase IV (Thermo Fisher, 17104019) and 40 U ml^−1^ DNase I. At the end of incubation, the suspension was homogenized with a 19-gauge syringe and filtered through a 70-µm strainer.

### Generation of bone marrow (competitive) chimeras

Eighteen-week-old CD45.2 or CD45.1/CD45.2 IM^DTR^ mice were anesthetized by i.p. injection of 200 µl PBS containing ketamine (75 mg per kg body weight; Dechra, 804132) and xylazine (10 mg per kg body weight; Bayer, 0076901). The thoracic cavity was protected with a 0.6-cm-thick lead cover and mice were lethally irradiated with two doses of 6 Gy 15 min apart. Once recovered from the anesthesia, mice were reconstituted by i.v. administration of 10^7^ BM cells from congenic CD45.1 wild-type mice. For mixed BM chimeras, mice were injected i.v. with 10^7^ BM cells consisting of a 1:1 mix of cells obtained from CD45.1 wild-type and CD45.2 *Ccr2*^−/−^, *Nr4a1*^−/−^ or *Ms4a3*^Cre^*Mafb*^fl/fl^ mice. From the day of irradiation, mice were treated for 4 weeks with 0.05 mg ml^−1^ of enrofloxacin (Baytril, Bayer) in drinking water. Chimerism was assessed by flow cytometry in the blood 4 weeks after irradiation.

### Adoptive transfer of bone marrow monocytes

BM Ly6C^+^ monocytes were isolated from congenic CD45.1 wild-type mice using the Monocyte Isolation Kit (Miltenyi Biotec, 130-100-629). Around 2 × 10^6^ BM Ly6C^+^ monocytes were administered i.v. into CD45.1/CD45.2 IM^DTR^ mice that were injected i.p. with 50 ng DT 24 h before monocyte transfer to deplete endogenous IMs.

### Flow cytometry

Cells (0.5–5 × 10^6^) were pre-incubated with Mouse BD Fc Block (BD Biosciences, 553142) to avoid unspecific binding to Fc receptors and stained with appropriate antibodies at 4 °C in the dark for 30 min. For EdU staining, extracellular-stained cells were permeabilized and stained using Click-iT EdU Alexa Fluor 488 Flow Cytometry Assay Kit (Thermo Fisher, 10632), according to the manufacturer’s instructions. For DAPI cell cycle analyses, extracellular-stained cells were permeabilized and stained with 1 µg ml^−1^ DAPI (BioLegend, 422801) in the dark for 30 min at room temperature (RT). For Ki67 stainings, extracellular-stained cells were permeabilized and stained using either FITC Mouse Anti-Ki67 Set (BD Biosciences, 556026) or PerCP-eFluor710 Mouse Anti-Ki67 (Thermo Fisher, 46-5698-80). Cell viability was assessed using LIVE/DEAD Fixable Near-IR (775) stain (Thermo Fisher, L34976) and the cell suspensions were analyzed with an LSRFortessa (BD Biosciences). Results were analyzed using FlowJo software (Tree Star). For scRNA-seq and bulk RNA-seq, lung myeloid cells were sorted using a FACSAria III (BD Biosciences). The full list of antibodies used can be found in the Supplementary Table 1.

### MCP-1/Ccl2 quantification

IM^DTR^ and littermate control mice were euthanized at indicated time points after DT administration. Blood was collected and lungs were perfused through the right ventricle with 10 ml PBS and isolated. Blood samples were left undisturbed for 30–45 min at RT to allow clot formation. The serum was separated from the blood clot by centrifugation for 10 min at 2,000g at 4 °C. Serum was stored at −80 °C. Dissected lungs were snap frozen and homogenized in 360 µl ice-cold lysis buffer (40 mM Tris-HCl (pH 7.4), 150 mM NaCl, 10% glycerol and cOmplete Protease Inhibitor Cocktail (Sigma, 11697498001) using a tissue homogenizer (IKA) with the addition of 1% NP-40 (Sigma, 74385) after homogenization. Samples were then rotated for 20 min at 4 °C, followed by a centrifugation to pellet debris. Protein concentration of cleared lysates was determined using Pierce BCA Protein Assay Kit (Thermo Fisher), according to the manufacturer’s instructions. Cleared lysates were stored at −80 °C. Ccl2 levels in serum and lung homogenates were determined using MCP-1/Ccl2 Mouse Uncoated ELISA Kit (Thermo Fisher), according to the manufacturer’s instructions.

### Bulk RNA-seq: sample preparation and analysis

Native IM subsets, cMo and AMs were isolated from uninjected IM^DTR^ mice, while repopulated IM subsets were isolated from IM^DTR^ mice that had been treated i.p. with 50 ng DT 14 d earlier. Cell populations were FACS sorted using the gating strategy shown in Fig. 1c into TRIzol reagent (Thermo Fisher, 10296010). Total RNA was extracted with the standard TRIzol RNA extraction protocol. RNA quality and quantity were evaluated using a 2100 bioanalyzer (Agilent) and the Quant-iT RiboGreen RNA Assay Kit (Thermo Fisher, R11490). One hundred nanograms of RNA was used to generate the libraries using the TruSeq Stranded mRNA kit (Illumina, 20020594). These libraries were sequenced on an Illumina NovaSeq sequencer on an SP flow cell. Sequence alignment with the mouse genome (GRCm38), sequence counting and quality control were performed using the nf-core/rnaseq pipeline. RNA-seq data were analyzed using R Bioconductor (3.5.1) and DESeq2 package (version 1.26.0)^50^.

### scRNA-seq

To compare lung monocytes and IMs from untreated IM^DTR^ mice (group ‘no treatment’) with those from IM^DTR^ mice treated with 50 ng DT i.p. 96 h before (group ‘DT96h’), five mice from each group were killed and lung single-cell suspensions were obtained after enzymatic digestion. CD11b^+^ cells were enriched by MACS using CD11b MicroBeads (Miltenyi Biotec, 130-049-601). Lung monocytes and IMs were then FACS sorted separately as CD45^+^SSC^lo^CD11b^+^F4/80^+^ CD64^−^ and CD64^+^ cells, respectively (Fig. 1c), and the 10x Genomics platform (Single Cell 3′ Solution) was used for scRNA-seq. The IM pool was then enriched in the final single-cell suspension to reach a monocyte/IM ratio of 1:1. For each sample, an aliquot of Trypan blue-treated cells was examined under the microscope for counting, viability and aggregate assessment following FACS sorting. Viability was above 90% for all samples and no aggregates were observed. Cell preparations were resuspended in calcium-free and magnesium-free PBS containing 0.4 mg ml^−1^ of UltraPure BSA (Thermo Fisher Scientific, AM2616).

To analyze lung monocytes (CD45^+^SSC^lo^CD11b^+^F4/80^+^CD64^−^) and IMs (CD45^+^SSC^lo^CD11b^+^F4/80^+^CD64^+^) from IM^DTR^ mice treated 12 h (group ‘DT12h’), 24 h (group ‘DT24’) and 48 h (group ‘DT48h’) before with 50 ng DT i.p., and to analyze lung monocytes (CD45^+^SSC^lo^CD11b^+^F4/80^+^CD64^−^) and CD64^+^ cells (CD45^+^SSC^lo^CD11b^+^F4/80^+^CD64^+^) from *Lyz2*^Cre^*Mafb*^fl/f/l^ (group ‘Mafb-KO’), *Lyz2*^Cre^*Maf*^fl/fl^ (group ‘cMAF-KO’) and littermate control (group ‘control’) mice, a similar protocol was applied, but cells from each group were barcoded with different anti-mouse Hashtag antibodies (BioLegend) before being pooled for encapsulation and library construction. To obtain a higher resolution in analyzing lung myeloid cells in myeloid-restricted *Mafb*-deficient and *Maf*-deficient mice, the pooled Mafb-KO/cMAF-KO/control samples were composed of a ratio of monocytes:CD64^+^ cells of 3:7 instead of 1:1.

For library preparation, approximately 3,000 cells per sample (for ‘DT96h’ and ‘no treatment’), or 20,000 cells for pooled hashtag-labeled samples, were loaded into the Chromium Controller, in which they were partitioned, and their polyA RNAs captured and barcoded using Chromium Single Cell 3′ GEM, Library & Gel Bead Kit v3 (10x Genomics). The cDNAs were amplified and libraries compatible with Illumina sequencers were generated using Chromium Single Cell 3′ GEM, Library & Gel Bead Kit v3 (10x Genomics). For Hash Tag Oligonucleotide (HTO) library, 1 µl HTO additive primer v2 (0.2 µM stock) were added to the mix at the cDNA amplification step. The libraries were sequenced on an Illumina NovaSeq sequencer on an SP100 cell flow (read 1, 28 cy; read 2, 76 cy; index 1, 10 cy; index 2, 10 cy) at a depth of 50,000 reads per cell.

The Cell Ranger (v3.0.2) application (10x Genomics) was then used to demultiplex the BCL files into FASTQ files (cellranger mkfastq), to perform alignment (to Cell Ranger human genome references 3.0.2 GRCm38/build 97), filtering and unique molecular identifier counting and to produce gene-barcode matrices (cellranger count).

Filtered matrix files were used for further scRNA-seq analyses with R Bioconductor (3.12) and Seurat (3.2.1)^51^. The cells from pooled hashtag-labeled samples were demultiplexed with the barcode detected in each cell.

Filtered matrices containing cell IDs and feature names in each sample were used to build a Seurat object. We performed quality control by filtering out the cells with less than 200 detected genes, the genes detected in less than three cells and the cells exhibiting more than 10% of mitochondrial genes. Gene counts in each sample were normalized separately by default method ‘LogNormalize’ with a scale factor of 10,000 and log transformation. Two thousand highly variable features were identified with the ‘vst’ method.

After merging cells from all samples, cell contaminants were removed based on the expression of specific genes. Four clusters were identified in the remaining cells using the FindClusters function and the DEGs were calculated using the FindAllMarkers function (Seurat package).

### Single-cell RNA velocity estimation

The counts for unspliced and ambiguous transcripts were calculated from CellRanger output using the velocyto command-line tool (http://velocyto.org/)^52^ and saved in loom files. The single-cell RNA velocities were estimated using scVelo toolkit (https://scvelo.readthedocs.io/)^53^. Briefly, the loom files were used as input for scVelo analysis. Genes with a minimum of 20 of both unspliced and spliced counts and on the top list of 2,000 genes were filtered, normalized and log transformed (scv.pp.filter_and_normalize with default parameters). Thirty principal components and 30 neighbors obtained from Euclidean distances in principal-component analysis space were used for computing first-order and second-order moments for each cell. We used generalized dynamical modeling to recover the full splicing kinetics of spliced genes, and the single-cell RNA velocities were plotted with the same cluster labels and embedding as in Fig. 4a.

### Gene Ontology enrichment analysis with differentially expressed gene signatures

The DEG lists for enrichment analyses were calculated using Seurat function FindMarkers with only.pos = TRUE to output only positively regulated genes. Thresholds logfc.threshold of 0.2 and adjusted *P* value of 0.01 were applied to filter the gene lists. Gene Ontology (GO) enrichment analyses were made using enrichGO functions from clusterProfiler package^54^ with default arguments. Only biology process terms of ontology were shown in the final results.

### Immunofluorescence

For lung immunofluorescence stainings, lungs were perfused with 10 ml PBS via the left ventricle and lungs were collected. Lungs were fixed for 4 h in 4% paraformaldehyde (Thermo Fisher, F/1501/PB15) at 4 °C. Fixed lungs were then cryoprotected in 30% sucrose (VWR, Avantor, 57-50-1) in PBS for 4 h at 4 °C, followed by embedding in optimal cutting temperature compound (OCT; Tissue-Tek, 4583) at −80 °C overnight, and lung OCT sections were cut (7-µm-thick sections) and blocked in methanol 100% (Merck, 67-56-1) at −20 °C for 20 min. Samples were stained in blocking buffer (PBS with 0.3% Triton X-100 (Merck, 648466) and 2% donkey serum (Sigma Aldrich, D9663)) with rat anti-mouse antibodies directed against MHC class II (I-A/I-E; 1:100 dilution in blocking buffer; clone M5/114.15.2, eBioscience, 14-5321-82) overnight at 4 °C. After washing samples with PBS, secondary donkey anti-rat IgG antibodies conjugated with Alexa Fluor 594 (1:1,000 dilution in blocking buffer; Thermo Fisher, A21209) were added in blocking buffer and incubated for 1 h in the dark at RT. Samples were washed with PBS and incubated with Alexa Fluor 488-conjugated rat anti-mouse antibodies directed against CD206 (clone C068C2, BioLegend, 141710; 1:50 dilution in blocking buffer), eFluor 570-conjugated rat anti-mouse antibodies directed against Ki67 (1:200 dilution in blocking buffer; clone SolA15, eBioscience, 41-5698-82), APC-conjugated rat anti-mouse antibodies directed against CD11b (1:50 dilution in blocking buffer; clone M1/70, eBioscience, 17-0112-83) in blocking buffer for 6 h at 4 °C. Finally, samples were washed one last time with PBS and were mounted with 10 μl ProLong Antifade reagent (Invitrogen, P36961) containing 0.1% Sytox blue nucleic acid stain (Invitrogen, S11348) on glass slides and stored at RT in the dark overnight.

All samples were analyzed by spectral fluorescence microscopy. Images of full lung sections were acquired on an LSM 980 with Airyscan 2 inverted confocal microscope (Zeiss) using a LD C-Apochromat ×40/1.1 W objective and Zen Black software. All fluorophores were excited simultaneously, and the emission spectra were collected with a spectral detector 32-channel GaAsP photomultiplier tube in lambda mode at 8.8-nm bins from 411 to 694 nm. A spectral unmixing was performed based on the monospectral spectra. Images were processed with the Zen Blue software. For quantification, the numbers of CD11b^+^CD206^lo^MHC-II^hi^ IMs (CD206^−^ IMs), CD11b^+^CD206^hi^MHC-II^lo/int^ IMs (CD206^+^ IM) and CD11b^+^CD206^−^MHC-II^lo^Ki67^hi^ cells were counted blindly and manually on a total surface of 12–16 mm^2^ per mouse section. The results were expressed in cell number per mm^2^ of lung section.

### Single-cell regulatory network inference and clustering analysis

To predict the potential active transcription factors, Ly6C^+^ cMo, Tr-Mo, CD206^−^ IMs and CD206^+^ IMs were subjected to SCENIC analysis using the SCENIC package^33^. The normalized counts, nFeature_RNA and nCount_RNA in merged Seurat object were used for the initial SCENIC analysis. The genes expressed with a value of 3 in 0.5% of the cells and detected in 1% of the cells were kept for following SCENIC analysis. Coexpression network analysis was made with GENIE3 in the SCENIC package. To represent the SCENIC results, the results of the ‘3.4_regulonAUC’ output were added to the metadata of Seurat object so that regulon AUC scores could be plotted using the FeaturePlot function. The top 50 regulons with highest variance are shown with their *z*-scores in the heat map.

### Monocle, tradeSeq and pseudotime analysis during interstitial macrophage development

To evaluate trajectory-based differential expression analysis during IM development in IM^DTR^ mice, Ly6C^+^ cMo, Tr-Mo, CD206^−^ IMs and CD206^+^ IMs were subjected to Monocle^28^ analysis. The Monocle CDS object was built with counts and metadata from Seurat object and converted using SeuratWrappers package. Cells were clustered with the cluster_cells function using calculated UMAP coordinates and a resolution of 0.51 × 10^−3^. The trajectories along pseudotime were built using learn_graph and order_cells functions. The DEGs across trajectories were calculated using Moran’s I test (graph_test function) and only the genes with a *q* value of 0 and Morans’s I value over 0.25 were kept as significant DEGs and subjected to further analyses.

To compare the expression patterns of DEGs across pseudotime, the counts matrix, pseudotime and cell weights calculated above were then used as input in fitGAM function (tradeSeq package)^29^. The association of average expression of each gene with pseudotime was tested using associationTest and the DEGs between CD206^−^ IM and CD206^+^ IM trajectories were calculated with the diffEndTest function. The value of the estimated smoother on a grid of pseudotimes was estimated for each DEG using predictSmooth. The DEG with waldStat > 70 and |log fold change| > 2 were annotated as ‘changed genes’, meaning that their expression patterns were different in CD206^−^ and CD206^+^ IM trajectories, while the rest of the DEGs were considered as ‘unchanged genes’, meaning that the expression patterns were similar in both trajectories. Finally, the scaled estimated smoothers calculated by predictSmooth were used to build heat maps with the ComplexHeatmap package^55^.

### Interstitial macrophage and monocyte signature scoring

The IM-specific, cMo-specific and CD16.2^+^ Mo-specific gene signatures were calculated using previously published scRNA-seq data^20^ by comparing IM, cMo or CD16.2^+^ Mo populations to all other cell types in the dataset using the FindMarker function (Seurat). The genes with |log fold change| > 1 and only positively regulated ones were considered as the IM, cMo or CD16.2^+^ Mo signature. The signatures were then used to calculate the scores for each cell using the VISION package^56^ (Fig. 8i) or with AddModuleScore function (Seurat; Extended Data Fig. 4e). The scores were stored in Seurat object and plotted with Seurat package.

### Statistical analysis

Graphs were prepared with Prism 9 (GraphPad) or R Bioconductor (3.5.1)^57^, and ggplot2 for data in Fig. 3b. No statistical methods were used to predetermine sample sizes, but our sample sizes are similar to those reported in previous publications^18,20,24,58^. Data distribution was assumed to be normal when parametric tests were performed, but this was not formally tested. Data from independent experiments were pooled for analysis in each data panel, unless otherwise indicated. No data were excluded from the analyses. Statistical analyses were performed with Prism 9 (GraphPad), and with R Bioconductor (3.5.1)^57^ and DESeq2 (ref. ^50^) or Seurat (3.2.1.)^51^ for bulk and scRNA-seq data, respectively. The statistical analyses performed for each experiment are indicated in the respective figure legends. We considered a *P* value lower than 0.05 to be significant (^*^, *P* < 0.05; ^**^, *P* < 0.01; ^***^, *P* < 0.001; ^****^, *P* < 0.0001).

### Reporting summary

Further information on research design is available in the Nature Portfolio Reporting Summary linked to this article.

## Online content

Any methods, additional references, Nature Portfolio reporting summaries, source data, extended data, supplementary information, acknowledgements, peer review information; details of author contributions and competing interests; and statements of data and code availability are available at 10.1038/s41590-023-01468-3.

## Supplementary information

Supplementary Information Supplementary Tables 1 and 2

Reporting Summary

## Data Availability

Single-cell RNA-seq and bulk RNA-seq data have been deposited at the Gene Expression Omnibus and are publicly available under accession GSE194021. A complete list of the data generated and used in this paper can be found in Supplementary Table 2. Source data are provided with this paper.

## Source data

Source Data Fig. 1 Statistical source data of panels b, e and g (raw data and *P* values).
Source Data Fig. 2 Statistical source data of panels d, e, f, g and h (raw data and *P* values).
Source Data Fig. 3 Statistical source data of panels b, c, e and g (raw data and *P* values).
Source Data Fig. 6 Statistical source data of panels d, e, g, i, k, l and n (raw data and *P* values).
Source Data Fig. 7 Statistical source data of panels d, e, g, j, k and l (raw data and *P* values).
Source Data Fig. 8 Statistical source data of panel g (raw data and *P* values).
Source Data Extended Data Fig. 2 Statistical source data of panels f–h (raw data and *P* values).
Source Data Extended Data Fig. 3 Statistical source data of panels c, e, g, i and j (raw data and *P* values).
Source Data Extended Data Fig. 4 Statistical source data of panels a and d (raw data and *P* values).
Source Data Extended Data Fig. 6 Statistical source data of panels a, b and e (raw data and *P* values).
Source Data Extended Data Fig. 7 Statistical source data of panel b (raw data and *P* values).
Source Data Extended Data Fig. 8 Statistical source data of panels d, f and j (raw data and *P* values).

## Code availability

All original codes have been deposited at GitHub and are available at: https://github.com/BlanQwall/Lung_IM_differentiation. Any additional information required to reanalyze the data reported in this paper is available from the corresponding author upon request.
