# Supplementary material for: MafB-restricted local monocyte proliferation precedes lung interstitial macrophage differentiation
Source: Nat Immunol. 2023 Mar 16;24(5):827–40. doi: 10.1038/s41590-023-01468-3 (PMC10154211; doi:10.1038/s41590-023-01468-3)
Supplement: Supplementary file 1 — Supplementary Tables 1 and 2 [file 41590_2023_1468_MOESM1_ESM.pdf]

nature immunology

Article https://doi.org/10.1038/s41590-023-01468-3

MafB-restricted local monocyte proliferation precedes lung interstitial macrophage differentiation

In the format provided by the
authors and unedited

Table of contents

Supplementary Tables

Supplementary Table 1. List of reagents, antibodies and commercial assays used in this study.

Supplementary Table 2. List of the deposited data generated and used in this study.

Supplementary Tables

Supplementary Table 1. List of reagents, antibodies and commercial assays used in this study.

Antibodies	Source	Cat. Number
Anti-Cre Recombinase Monoclonal Antibody (Rabbit, clone D7L7L), unconjugated	Cell Signaling Technology	15036
Anti-mouse C1qA Monoclonal Antibody (Mouse, clone JL-1), biotin conjugated	Bio-techne	NBP1-51140B
Anti-mouse CD3 Monoclonal Antibody (Rat, clone 17A2), eFluor450 conjugated	ThermoFisher	48-0032-82
Anti-mouse CD3e Monoclonal Antibody (Armenian Hamster, clone 145-2C11), PE conjugated	BD Biosciences	553064
Anti-mouse CD11b Monoclonal Antibody (Rat, clone M1/70), APC conjugated	ThermoFisher	17-0112-82
Anti-mouse CD11b Monoclonal Antibody (Rat, clone M1/70), BUV395 conjugated	BD Biosciences	563553
Anti-mouse CD11b Monoclonal Antibody (Rat, clone M1/70), BUV563 conjugated	BD Biosciences	741242
Anti-mouse CD11b Monoclonal Antibody (Rat, clone M1/70), FITC conjugated	BD Biosciences	557396
Anti-mouse CD11b Monoclonal Antibody (Rat, clone M1/70), PE-Cy7 conjugated	BD Biosciences	552850
Anti-mouse CD11c Monoclonal Antibody (Hamster, clone HL3), BV786 conjugated	BD Biosciences	563735
Anti-mouse CD16/32 (Mouse BD Fc Block™) Monoclonal Antibody (Rat, clone 2.4G2), unconjugated	BD Biosciences	553142
Anti-mouse CD16/32 Monoclonal Antibody (Rat, clone 2.4G2), APC-Cy7 conjugated	BD Biosciences	560541
Anti-mouse CD19 Monoclonal Antibody (Rat, clone 1D3), PE conjugated	BD Biosciences	553786
Anti-mouse CD31 Monoclonal Antibody (Rat, clone 390), APC conjugated	ThermoFisher	17-0311-82
Anti-mouse CD34 Monoclonal Antibody (Rat, clone SA376A4), BV421 conjugated	BioLegend	152207
Anti-mouse CD45.1 Monoclonal Antibody (Mouse, clone A20), BUV395 conjugated	BD Biosciences	565212
Anti-mouse CD45.1 Monoclonal Antibody (Mouse, clone A20), BV510 conjugated	BD Biosciences	565278
Anti-mouse CD45.2 Monoclonal Antibody (Mouse, clone 104), BUV395 conjugated	BD Biosciences	564616
Anti-mouse CD45.2 Monoclonal Antibody (Mouse, clone 104), FITC conjugated	BD Biosciences	561874
Anti-mouse CD45.2 Monoclonal Antibody (Mouse, clone 104), PE-Cy7 conjugated	BD Biosciences	560696

Anti-mouse CD45.2 Monoclonal Antibody (Mouse, clone 104), PerCP-Cy5.5 conjugated	BD Biosciences	552950
Anti-mouse CD45.2 Monoclonal Antibody (Mouse, clone 104), V500 conjugated	BD Biosciences	562129
Anti-mouse CD64 Monoclonal Antibody (Mouse, clone X54-5/7.1), BV421 conjugated	BioLegend	139309
Anti-mouse CD115 (CSF1R) Monoclonal Antibody (Rat, clone AFS98), unconjugated	Bio X Cell	BE0213
Anti-mouse CD115 (CSF1R) Monoclonal Antibody (Rat, clone AFS98), APC conjugated	ThermoFisher	17-1152-82
Anti-mouse CD115 (CSF1R) Monoclonal Antibody (Rat, clone AFS98), PerCP-Cy5.5 conjugated	BioLegend	135526
Anti-mouse CD117 (c-Kit) Monoclonal Antibody (Rat, 2B8), BV786 conjugated	BD Biosciences	564012
Anti-mouse CD117 (c-Kit) Monoclonal Antibody (Rat, 2B8), PE conjugated	BioLegend	105807
Anti-mouse CD135 (Flt3) Monoclonal Antibody (Rat, clone A2F10), PE conjugated	ThermoFisher	12-1351-82
Anti-mouse CD170 (SiglecF) Monoclonal Antibody (Rat, clone E50-2440), PE conjugated	BD Biosciences	552126
Anti-mouse CD170 (SiglecF) Monoclonal Antibody (Rat, clone E50-2440), PE-CF594 conjugated	BD Biosciences	562757
Anti-mouse CD172a (SIRPa) Monoclonal Antibody (Rat, clone P84), APC conjugated	BioLegend	144013
Anti-mouse CD206 (MMR) Monoclonal Antibody (Rat, clone C068C2), AF647 conjugated	BioLegend	141712
Anti-mouse CD206 (MMR) Monoclonal Antibody (Rat, clone C068C2), AF647 conjugated	BioLegend	141712
Anti-mouse CD206 (MMR) Monoclonal Antibody (Rat, clone C068C2), PE-Cy7 conjugated	BioLegend	141719
Anti-mouse cMaf Monoclonal Antibody (Mouse, clone sym0F1), PE conjugated	ThermoFisher	12-9855-42
Anti-mouse F4/80 Monoclonal Antibody (Rat, BM8), BV650 conjugated	BioLegend	123149
Anti-mouse F4/80 Monoclonal Antibody (Rat, BM8), FITC conjugated	BioLegend	123108
Anti-mouse F4/80 Monoclonal Antibody (Rat, BM8), PE conjugated	Sony Biotechnology	1215550
Anti-mouse FcεRIα (MAR-1) Monoclonal Antibody (Armenian Hamster, clone MAR-1), PE-Cy7 conjugated	BioLegend	134317
Anti-mouse I-A/I-E (MHC-II) Monoclonal Antibody (Rat, clone M5/114.15.2), unconjugated	ThermoFisher	56-5321-82

Anti-mouse I-A/I-E (MHC-II) Monoclonal Antibody (Rat, clone M5/114.15.2), AF700 conjugated	ThermoFisher	56-5321-80
Anti-mouse I-A/I-E (MHC-II) Monoclonal Antibody (Rat, clone M5/114.15.2), PerCP-Cy5.5 conjugated	Sony Biotechnology	1138125
Anti-mouse Ki-67 Monoclonal Antibody (Rat, clone SolA15), eFluor570 conjugated	ThermoFisher	41-5698-82
Anti-mouse Ki-67 Monoclonal Antibody (Rat, clone SolA15), PerCP-eFluor710 conjugated	ThermoFisher	46-5698-80
Anti-mouse Ly6A/E Monoclonal Antibody (Rat, clone D7), PE-Cy7 conjugated	BD Biosciences	561021
Anti-mouse Ly6C Monoclonal Antibody (Rat, clone HK1.4), AF700 conjugated	BioLegend	128024
Anti-mouse Ly6C Monoclonal Antibody (Rat, clone AL-21), PE-CF594 conjugated	BD Biosciences	562728
Anti-mouse Ly6G Monoclonal Antibody (Rat, clone 1A8), APC conjugated	BD Biosciences	560599
Anti-mouse Ly6G Monoclonal Antibody (Rat, clone 1A8), FITC conjugated	BD Biosciences	551461
Anti-mouse Ly6G Monoclonal Antibody (Rat, clone 1A8), PE conjugated	BD Biosciences	551461
Anti-mouse Ly6G Monoclonal Antibody (Rat, clone 1A8), PE-Cy7 conjugated	BD Biosciences	560601
Anti-mouse Ly6G Monoclonal Antibody (Rat, clone 1A8), PerCP-Cy5.5 conjugated	BioLegend	127615
Anti-mouse MafB Recombinant Monoclonal Antibody (Rabbit, clone BLR046F), unconjugated	Bethyl Laboratories Inc.	A700-046
Anti-mouse MerTK Monoclonal Antibody (Rat, clone DS5MMER), PE-Cy7 conjugated	ThermoFisher	25-5751-80
Anti-mouse XCR-1 Monoclonal Antibody (Mouse, clone ZET), APC-Cy7 conjugated	BioLegend	148223
Anti-rabbit IgG (H+L) Cross-Adsorbed Secondary Polyclonal Antibody (Goat), AF488 conjugated	ThermoFisher	A-11008
Anti-rabbit IgG (H+L) Cross-Adsorbed Secondary Polyclonal Antibody (Goat), AF647 conjugated	ThermoFisher	A-21244
Anti-rat IgG (H+L) Cross-Adsorbed Secondary Polyclonal Antibody (Donkey), AF594 conjugated	ThermoFisher	A-21209
Anti-trinitrophenol (isotype control) Monoclonal Antibody (Rat, 2A3), unconjugated	Bio X Cell	BE0089
TotalSeq™-A0305 anti-mouse Hashtag 5 Antibody	BioLegend	155809
TotalSeq™-A0306 anti-mouse Hashtag 6 Antibody	BioLegend	155811
TotalSeq™-A0307 anti-mouse Hashtag 7 Antibody	BioLegend	155813

TotalSeq™-A0308 anti-mouse Hashtag 8 Antibody	BioLegend	155815
**Chemicals, Peptides and Recombinant Proteins**	**Source**	**Cat. Number**
1,4 Dithiothreitol (DTT)	Sigma	10197777001
5-Ethynyl-2′-deoxyuridine (EdU)	Santa Cruz Biotechnology	sc-284628
Baytrill (enrofloxacine)	Bayer	616300
Bovine Serum Albumin (BSA)	Sigma	A7906
Brilliant Stain Buffer	BD Bioscience	563794
Collagenase A, from *Clostridium histolyticum*	Sigma	11088793001
Collagenase IV	ThermoFisher	17104019
cOmplete™ Protease Inhibitor Cocktail	Sigma	11697498001
Diphtheria Toxin (DT), from *Corynebacterium diphtheriae*	List Biological Labs	150
DAPI	Biolegend	422801
DNase I	Sigma	11284932001
dNTP	ThermoFisher	N8080260
Donkey serum	Sigma	D9663
DPBS	ThermoFisher	14190094
EDTA	Merck Millipore	1084181000
Fetal Bovine Serum (FBS)	ThermoFisher	10270098
Glycerol	ThermoFisher	158920025
GoTaq G2 Hot Start Taq Polymerase	Promega	M7401
HBSS with Phenol Red	Lonza	BE10-508F
IsoFlo (Isoflurane)	Zoetis	B506
KAPA Express Extract	Merck	KK7100
Methanol	Merck	67-56-1
Nimatek (Ketamine)	Dechra	804132
Nonidet P 40 Substitute	Sigma	74385
O.C.T. Compound	Tissue-Tek	4583
Paraformaldehyde	ThermoFisher	F/1501/PB15
Percoll	GE Healthcare	17089101
Pexidartinib (PLX-3397)	MedChemExpress	HY-16749
Poly(ethylene glycol), average Mn 400	Sigma	202398
ProLong Antifade Mountant	ThermoFisher	P36961
Quant-iT™ RiboGreen™ RNA Assay Kit	ThermoFisher	R11490
Rompun (Xylazine)	Bayer	0076901
Sucrose	Merck	57-50-1
Streptavidin, PE conjugated	BD Biosciences	554061
SYTOX Blue Nucleic Acid Stain	ThermoFisher	S11348
Tris(hydroxymethyl)aminomethane	Merck Millipore	108382
TRIzol Reagent	ThermoFisher	10296010
Triton X-100	Merck	648466
Truseq Stranded mRNA kit	Illumina	20020594
Tween-20	ThermoFisher	233360010
Tween-80	Sigma	P1754
G418	ThermoFisher	10131027
UltraPure BSA	ThermoFisher	AM2616
**Commercial Assays**	**Source**	**Cat. Number**

APC Mouse Lineage Antibody Cocktail, with Isotype Control	BD Bioscience	558074
CD11b MicroBeads, human and mouse	Miltenyi Biotec	130-049-601
Chromium Next GEM Single Cell 3' GEM, Library & Gel Bead Kit v3.1	10X Genomics	1000128
CD11b MicroBeads, human and mouse	Miltenyi Biotec	130-049-601
Chromium Next GEM Single Cell 3' GEM, Library & Gel Bead Kit v3.1	10X Genomics	1000128
Chromium Next GEM Chip G Single Cell Kit	10X Genomics	1000120
Click-iT™ Plus EdU Alexa Fluor™ 488 Flow Cytometry Assay Kit	ThermoFisher	10632
FITC Mouse Anti-Ki-67 Set	BD Bioscience	556026
LIVE/DEAD™ Fixable Near-IR Dead Cell Stain Kit, for 633 or 635 nm excitation	Invitrogen	L34976
MCP-1/CCL2 Mouse Uncoated ELISA Kit	ThermoFisher	88-7391-88
Monocyte Isolation Kit (BM), mouse	Miltenyi Biotec	130-100-629
Pierce™ BCA Protein Assay Kit	ThermoFisher	23225
Single Index Kit T Set A	10X Genomics	1000213
NovaSeq 6000 S1 Reagent Kit v1.5 (100 cycles)	Illumina	20028319

Supplementary Table 2. List of the deposited data generated and used in this study.

Bulk RNA-seq data	Source	Identifier
Raw and analyzed RNA-seq data	This paper	GEO: GSE194021
**scRNA-seq data**	**Source**	**Identifier**
Raw and analyzed scRNA-seq data	This paper	GEO: GSE194021
Lung IM and classical monocytes	[19]	EMBL-EBI: E-MTAB-7678
**Microarray data**	**Source**	**Identifier**
Classical Monocytes MHCII$^+$ in blood	ImmGen	GEO: GSM605868, GSM605870, GSM605871
Classical Monocytes MHCII$^-$ in bone marrow	ImmGen	GEO: GSM854329, GSM854330, GSM854331
Classical Monocytes MHCII$^-$ in blood	ImmGen	GEO: GSM605872, GSM605873, GSM605874
Nonclassical Monocytes, MHCII$^+$	ImmGen	GEO: GSM605878, GSM605879
Nonclassical Monocytes in bone marrow	ImmGen	GEO: GSM854332, GSM854333, GSM854334
Nonclassical Monocytes in blood	ImmGen	GEO: GSM605884, GSM605885
Nonclassical Monocytes, MHCII^{int}	ImmGen	GEO: GSM605886, GSM605887, GSM605888, GSM605889, GSM605890
Lung CD11b$^+$CD24$^-$ macrophages	ImmGen	GEO: GSM854271, GSM854272
Small Intestinal Lamina Propria CD11c^{hi}CD103$^-$CD11b$^+$ macrophages	ImmGen	GEO: GSM854262, GSM854263, GSM854264, GSM854265, GSM854266, GSM854267, GSM854268
Bone marrow macrophages	ImmGen	GEO: GSM854317, GSM854318, GSM854319
Spleen Red Pulp macrophages	ImmGen	GEO: GSM605853, GSM605854, GSM605855
Peritoneal macrophage steady state	ImmGen	GEO: GSM854294, GSM854295, GSM854296
Peritoneal cavity macrophages steady state	ImmGen	GEO: GSM605850, GSM605851, GSM605852
Medullary macrophages from skin draining lymph nodes	ImmGen	GEO: GSM854322, GSM854323
Central nervous system microglia	ImmGen	GEO: GSM854326, GSM854327, GSM854328
CD103$^+$ migratory DC, Mediastinal LN CD103$^+$ DC	ImmGen	GEO: GSM854243, GSM854244, GSM854245
CD11b$^+$ migratory DC, Mediastinal LN CD11b$^+$ DC	ImmGen	GEO: GSM854255, GSM854256, GSM854257
Lung CD103$^+$ dendritic cells	ImmGen	GEO: GSM538231, GSM538232, GSM538233, GSM854241, GSM854242
Lung MHCII$^+$CD11c$^+$CD103$^-$ CD11b$^+$CD24$^+$ dendritic cells	ImmGen	GEO: GSM854269, GSM854270
Lung IM, Ly6C$^+$ cMo and AM	[22]	EMBL-EBI: E-MTAB-5012
