# Supplementary material for: MafB-restricted local monocyte proliferation precedes lung interstitial macrophage differentiation
Source: Nat Immunol. 2023 Mar 16;24(5):827–40. doi: 10.1038/s41590-023-01468-3 (PMC10154211; doi:10.1038/s41590-023-01468-3)
Supplement: Supplementary file 2 — Reporting Summary [file 41590_2023_1468_MOESM2_ESM.pdf]

nature portfolio

Corresponding author(s): Thomas Marichal
Last updated by author(s): Feb 14, 2023

Reporting Summary

Nature Portfolio wishes to improve the reproducibility of the work that we publish. This form provides structure for consistency and transparency in reporting. For further information on Nature Portfolio policies, see our Editorial Policies and the Editorial Policy Checklist.

Statistics

For all statistical analyses, confirm that the following items are present in the figure legend, table legend, main text, or Methods section.

n/a	Confirmed	
☐	☒	The exact sample size (*n*) for each experimental group/condition, given as a discrete number and unit of measurement
☐	☒	A statement on whether measurements were taken from distinct samples or whether the same sample was measured repeatedly
☐	☒	The statistical test(s) used AND whether they are one- or two-sided<br>*Only common tests should be described solely by name; describe more complex techniques in the Methods section.*
☒	☐	A description of all covariates tested
☐	☒	A description of any assumptions or corrections, such as tests of normality and adjustment for multiple comparisons
☐	☒	A full description of the statistical parameters including central tendency (e.g. means) or other basic estimates (e.g. regression coefficient) AND variation (e.g. standard deviation) or associated estimates of uncertainty (e.g. confidence intervals)
☐	☒	For null hypothesis testing, the test statistic (e.g. *F*, *t*, *r*) with confidence intervals, effect sizes, degrees of freedom and *P* value noted<br>*Give P values as exact values whenever suitable.*
☒	☐	For Bayesian analysis, information on the choice of priors and Markov chain Monte Carlo settings
☒	☐	For hierarchical and complex designs, identification of the appropriate level for tests and full reporting of outcomes
☒	☐	Estimates of effect sizes (e.g. Cohen's *d*, Pearson's *r*), indicating how they were calculated

Our web collection on statistics for biologists contains articles on many of the points above.

Software and code

Policy information about availability of computer code

Data collection	Bulk RNA-seq: Sequence alignment with the mouse genome (GRCm38), sequence counting and quality control were performed using the nfcore/rnaseq pipeline.<br>scRNA-seq: Cell Ranger (v3.0.2) (10x Genomics) was then used to demultiplex the BCL files into FASTQ files (cellranger mkfastq), to perform alignment (to Cell Ranger human genome references 3.0.2 GRCm38/build 97), filtering, UMI counting and to produce gene barcode matrices (cellranger count).<br>Flow cytometry: BD FACSDiva 7 and 8 (BD Biosciences)<br>Microscopy: ZEN Black (Zeiss)
Data analysis	RNA-seq data were analyzed using R Bioconductor (3.5.1) and DESeq2 package (version 1.26.0). scRNAseq analyses were performed with R Bioconductor (3.12), Seurat (3.2.1), velocyto (0.6), SCENIC (1.2.4), Monocle3 (1.0.0), tradeSeq (1.4.0), VISION (2.1.0) and clusterProfiler (3.18.1). All original codes have been deposited at GitHub and are available via this link: https://github.com/BlanQwall/Lung_IM_differentiation.<br>Flow cytometry: FlowJo 10 (TreeStar)<br>Microscopy: ZEN Blue (Zeiss)<br>Statistics: Prism 8 (GraphPad) & R Bioconductor using packages DESeq2, Seurat, tradeSeq and multcomp.

For manuscripts utilizing custom algorithms or software that are central to the research but not yet described in published literature, software must be made available to editors and reviewers. We strongly encourage code deposition in a community repository (e.g. GitHub). See the Nature Portfolio guidelines for submitting code & software for further information.

Data

Policy information about availability of data

All manuscripts must include a data availability statement. This statement should provide the following information, where applicable:

- Accession codes, unique identifiers, or web links for publicly available datasets
- A description of any restrictions on data availability
- For clinical datasets or third party data, please ensure that the statement adheres to our policy

Single-cell RNA-seq and bulk RNA-seq data have been deposited at GEO and are publicly available under GEO accession GSE 194021.
All original codes have been deposited at GitHub and are available via this link: https://github.com/BlanQwall/Lung_IM_differentiation.
Any additional information required to reanalyze the data reported in this paper is available from the corresponding author upon request.

Human research participants

Policy information about studies involving human research participants and Sex and Gender in Research.

Reporting on sex and gender	n/a
Population characteristics	n/a
Recruitment	n/a
Ethics oversight	n/a

Note that full information on the approval of the study protocol must also be provided in the manuscript.

Field-specific reporting

Please select the one below that is the best fit for your research. If you are not sure, read the appropriate sections before making your selection.

☒ Life sciences ☐ Behavioural & social sciences ☐ Ecological, evolutionary & environmental sciences

For a reference copy of the document with all sections, see nature.com/documents/nr-reporting-summary-flat.pdf

Life sciences study design

All studies must disclose on these points even when the disclosure is negative.

Sample size	No statistical methods were used to pre-determine sample sizes but our sample sizes are similar to those reported in previous publications (PMID: 28329706; PMID: 31481690; PMID: 31591573). Data distribution was assumed to be normal when parametric tests were performed, but this was not formally tested.
Data exclusions	No data were excluded from the analyses.
Replication	For each experiment, each experimental group was composed of 3-5 mice constituting biological replicates. Each experiments have been repeated two to four times. All attempts at replication were successful and gave similar readout.
Randomization	Mice were identified according to genotype and all experiments were performed with age- and sex-matched littermates. For Csf1r blocking experiments mice were randomly assigned to vehicle or isotype Ab and anti-Csf1r treatments. For experiments using IMDTR mice that were treated or not with DT, mice were randomly allocated to DT treatment or not.
Blinding	Investigators were not blinded during the collection and analysis of the data, except for the quantification of microscopy lung sections, where investigators were blinded.

Reporting for specific materials, systems and methods

We require information from authors about some types of materials, experimental systems and methods used in many studies. Here, indicate whether each material, system or method listed is relevant to your study. If you are not sure if a list item applies to your research, read the appropriate section before selecting a response.

Materials & experimental systems

n/a	Involved in the study
☐	☒ Antibodies
☒	☐ Eukaryotic cell lines
☒	☐ Palaeontology and archaeology
☐	☒ Animals and other organisms
☒	☐ Clinical data
☒	☐ Dual use research of concern

Methods

n/a	Involved in the study
☒	☐ ChIP-seq
☐	☒ Flow cytometry
☒	☐ MRI-based neuroimaging

Antibodies

Antibodies used

The following antibodies were used for flow cytometry.
Anti-Cre Recombinase Monoclonal Antibody (Rabbit, clone D7L7L), unconjugated;Cell Signaling Technology;15036
Anti-mouse C1qA Monoclonal Antibody (Mouse, clone JL-1), biotin conjugated;Bio-techne;NBP1-51140B
Anti-mouse CD3 Monoclonal Antibody (Rat, clone 17A2), eFluor450 conjugated ;ThermoFisher;48-0032-82
Anti-mouse CD3e Monoclonal Antibody (Armenian Hamster, clone 145-2C11), PE conjugated;BD Biosciences;553064
Anti-mouse CD11b Monoclonal Antibody (Rat, clone M1/70), BUV395 conjugated;BD Biosciences;563553
Anti-mouse CD11b Monoclonal Antibody (Rat, clone M1/70), BUV563 conjugated;BD Biosciences;741242
Anti-mouse CD11b Monoclonal Antibody (Rat, clone M1/70), FITC conjugated;BD Biosciences;557396
Anti-mouse CD11b Monoclonal Antibody (Rat, clone M1/70), PE-Cy7 conjugated;BD Biosciences;552850
Anti-mouse CD11c Monoclonal Antibody (Hamster, clone HL3), BV786 conjugated;BD Biosciences;563735
Anti-mouse CD16/32 (Mouse BD Fc Block™) Monoclonal Antibody (Rat, clone 2.4G2), unconjugated;BD Biosciences;553142
Anti-mouse CD16/32 Monoclonal Antibody (Rat, clone 2.4G2), APC-Cy7 conjugated;BD Biosciences;560541
Anti-mouse CD19 Monoclonal Antibody (Rat, clone 1D3), PE conjugated;BD Biosciences;553786
Anti-mouse CD31 Monoclonal Antibody (Rat, clone 390), APC conjugated;ThermoFisher;17-0311-82
Anti-mouse CD34 Monoclonal Antibody (Rat, clone SA376A4), BV421 conjugated;BioLegend;152207
Anti-mouse CD45.1 Monoclonal Antibody (Mouse, clone A20), BUV395 conjugated;BD Biosciences;565212
Anti-mouse CD45.1 Monoclonal Antibody (Mouse, clone A20), BV510 conjugated;BD Biosciences;565278
Anti-mouse CD45.2 Monoclonal Antibody (Mouse, clone 104), BUV395 conjugated;BD Biosciences;564616
Anti-mouse CD45.2 Monoclonal Antibody (Mouse, clone 104), FITC conjugated;BD Biosciences;561874
Anti-mouse CD45.2 Monoclonal Antibody (Mouse, clone 104), PE-Cy7 conjugated;BD Biosciences;560696
Anti-mouse CD45.2 Monoclonal Antibody (Mouse, clone 104), PerCP-Cy5.5 conjugated;BD Biosciences;552950
Anti-mouse CD45.2 Monoclonal Antibody (Mouse, clone 104), V500 conjugated;BD Biosciences;562129
Anti-mouse CD64 Monoclonal Antibody (Mouse, clone X54-5/7.1), BV421 conjugated;BioLegend;139309
Anti-mouse CD115 (CSF1R) Monoclonal Antibody (Rat, clone AFS98), APC conjugated;ThermoFisher;17-1152-82
Anti-mouse CD115 (CSF1R) Monoclonal Antibody (Rat, clone AFS98), PerCP-Cy5.5 conjugated;BioLegend;135526
Anti-mouse CD117 (c-Kit) Monoclonal Antibody (Rat, 2B8), BV786 conjugated;BD Biosciences;564012
Anti-mouse CD117 (c-Kit) Monoclonal Antibody (Rat, 2B8), PE conjugated;BioLegend;105807
Anti-mouse CD135 (Flt3) Monoclonal Antibody (Rat, clone A2F10), PE conjugated;ThermoFisher;12-1351-82
Anti-mouse CD170 (SiglecF) Monoclonal Antibody (Rat, clone E50-2440), PE conjugated;BD Biosciences;552126
Anti-mouse CD170 (SiglecF) Monoclonal Antibody (Rat, clone E50-2440), PE-CF594 conjugated;BD Biosciences;562757
Anti-mouse CD172a (SIRPa) Monoclonal Antibody (Rat, clone P84), APC conjugated;BioLegend;144013
Anti-mouse CD206 (MMR) Monoclonal Antibody (Rat, clone C068C2), AF647 conjugated;BioLegend;141712
Anti-mouse CD206 (MMR) Monoclonal Antibody (Rat, clone C068C2), PE-Cy7 conjugated;BioLegend;141719
Anti-mouse cMaf Monoclonal Antibody (Mouse, clone sym0F1), PE conjugated;ThermoFisher;12-9855-42
Anti-mouse F4/80 Monoclonal Antibody (Rat, BM8), BV650 conjugated;BioLegend ;123149
Anti-mouse F4/80 Monoclonal Antibody (Rat, BM8), FITC conjugated;BioLegend ;123108
Anti-mouse F4/80 Monoclonal Antibody (Rat, BM8), PE conjugated;Sony Biotechnology;1215550
Anti-mouse Fc?RI? (MAR-1) Monoclonal Antibody (Armenian Hamster, clone MAR-1), PE-Cy7 conjugated;BioLegend;134317
Anti-mouse I-A/I-E (MHC-II) Monoclonal Antibody (Rat, clone M5/114.15.2), AF700 conjugated;ThermoFisher;56-5321-80
Anti-mouse I-A/I-E (MHC-II) Monoclonal Antibody (Rat, clone M5/114.15.2), PerCP-Cy5.5 conjugated;Sony Biotechnology;1138125
Anti-mouse Ki-67 Monoclonal Antibody (Rat, clone SolA15), PerCP-eFluor710 conjugated;ThermoFisher;46-5698-80
Anti-mouse Ly6A/E Monoclonal Antibody (Rat, clone D7), PE-Cy7 conjugated;BD Biosciences;561021
Anti-mouse Ly6C Monoclonal Antibody (Rat, clone HK1.4), AF700 conjugated;BioLegend;128024
Anti-mouse Ly6C Monoclonal Antibody (Rat, clone AL-21), PE-CF594 conjugated;BD Biosciences;562728
Anti-mouse Ly6G Monoclonal Antibody (Rat, clone 1A8), APC conjugated;BD Biosciences;560599
Anti-mouse Ly6G Monoclonal Antibody (Rat, clone 1A8), FITC conjugated;BD Biosciences;551461
Anti-mouse Ly6G Monoclonal Antibody (Rat, clone 1A8), PE conjugated;BD Biosciences;551461
Anti-mouse Ly6G Monoclonal Antibody (Rat, clone 1A8), PE-Cy7 conjugated;BD Biosciences;560601
Anti-mouse Ly6G Monoclonal Antibody (Rat, clone 1A8), PerCP-Cy5.5 conjugated;BioLegend;127615
Anti-mouse MafB Recombinant Monoclonal Antibody (Rabbit, clone BLR046F), unconjugated;Bethyl Laboratories Inc.;A700-046
Anti-mouse MerTK Monoclonal Antibody (Rat, clone DS5MMER), PE-Cy7 conjugated;ThermoFisher;25-5751-80
Anti-mouse XCR-1 Monoclonal Antibody (Mouse, clone ZET), APC-Cy7 conjugated;BioLegend;148223
Anti-rabbit IgG (H+L) Cross-Adsorbed Secondary Polyclonal Antibody (Goat), AF488 conjugated;ThermoFisher;A-11008
Anti-rabbit IgG (H+L) Cross-Adsorbed Secondary Polyclonal Antibody (Goat), AF647 conjugated;ThermoFisher;A-21244
All antibodies were used at 1:200 dilution, except for C1qA, MafB and cMaf were used at 1:50 dilution, and anti-rabbit IgGs were used at 1:800 dilution.

The following antibodies were used for barcoding cells.
TotalSeq™-A0305 anti-mouse Hashtag 5 Antibody;BioLegend;155809

TotalSeq™-A0306 anti-mouse Hashtag 6 Antibody;BioLegend;155811
TotalSeq™-A0307 anti-mouse Hashtag 7 Antibody;BioLegend;155813
TotalSeq™-A0308 anti-mouse Hashtag 8 Antibody;BioLegend;155815
All antibodies were used at 1:100 dilution as recommended by BioLegend.

The following antibodies were used for immunofluorescence microscopy.
Unconjugated rat anti-mouse I-A/I-E (MHC-II) Monoclonal Antibody (clone M5/114.15.2; ThermoFisher; 56-5321-82; 1:100 dilution)
AF594 conjugated donkey anti-rat IgG (H+L) Cross-Adsorbed Secondary Polyclonal Antibody (ThermoFisher; A-21209; 1:1000 dilution)
AF488 conjugated rat anti-mouse CD206 (MMR) Monoclonal Antibody (clone C068C2; BioLegend; 141710; 1:50 dilution)
eFluor570 conjugated rat anti-mouse Ki-67 Monoclonal Antibody (clone SolA15; ThermoFisher; 41-5698-82; 1:200 dilution)
APC conjugated rat anti-mouse CD11b Monoclonal Antibody (clone M1/70; ThermoFisher; 17-0112-82; 1:50 dilution)

For Csf1r blocking experiments.
Unconjugated rat anti-mouse CD115 (CSF1R) Monoclonal Antibody (clone AFS98; Bio X Cell; BE0213)
Unconjugated rat anti-trinitrophenol (isotype control) Monoclonal Antibody (clone 2A3;Bio X Cell;BE0089)
Mice were injected i.v. with 250µg of anti-mouse Csf1r-blocking antibody (Clone AFS98, Bio X Cell, Cat#BE0213) or isotype control (Clone 2A3, Bio X Cell, Cat#BE0089) 6 and 28 h post-DT injection.

Validation

All the antibodies are from commercial sources that perform rigorous testing for specificity, quality control and lot to lot variability. Specific statements of validation include:

BD bioscience
https://www.bdbiosciences.com/en-us/products/reagents/flow-cytometry-reagents/research-reagents/quality-and-reproducibility
The specificity is confirmed using multiple methodologies that may include a combination of flow cytometry, immunofluorescence, immunohistochemistry or western blot to test staining on a combination of primary cells, cell lines or transfectant models. The manufacturing process for the BD Biosciences antibodies and reagents adheres to standard operating procedures (SOPs) and guidelines to ensure lot-to-lot consistency. All flow cytometry reagents are titrated on the relevant positive or negative cells.

BioLegend
https://www.biolegend.com/en-us/quality/quality-control
Flow cytometry reagents: Specificity testing of 1-3 target cell types with either single- or multi-color analysis (including positive and negative cell types). Once specificity is confirmed, each new lot must perform with similar intensity to the in-date reference lot. Brightness (MFI) is evaluated from both positive and negative populations. Each lot product is validated by QC testing with a series of titration dilutions.
TotalSeq Antibodies: Bulk lots are tested by PCR and sequencing to confirm the oligonucleotide barcodes. They are also tested by flow cytometry to ensure the antibodies recognize the proper cell populations. Bottled lots are tested by PCR and sequencing to confirm the oligonucleotide barcodes.

ThermoFisher
https://www.thermofisher.com/be/en/home/life-science/antibodies/invitrogen-antibody-validation.html
Invitrogen antibodies are currently undergoing a rigorous two-part testing approach. Part 1: Target specificity verification; Part 2: Functional application validation.

Sony Biotechnology
Each lot of each antibody is quality control tested by immunofluorescent staining with flow cytometric analysis.
PE conjugated anti-mouse F4/80 Monoclonal Antibody (Rat, BM8) was validated by flow cytometry on thioglycolate-elicited BALB/c mouse peritoneal macrophages.
PerCP-Cy5.5 conjugated anti-mouse I-A/I-E (MHC-II) Monoclonal Antibody (Rat, clone M5/114.15.2) was validated by flow cytometry on C57BL/6 mouse splenocytes.

Cell Signaling Technology
Anti-Cre Recombinase Monoclonal Antibody (Rabbit, clone D7L7L) was validated by Western blot analysis of extracts from 293T cells, mock-transfected or transfected with a construct expressing Cre recombinase.

Bethyl Laboratories
https://www.fortislife.com/antibody-validation
Bethyl antibodies are highly regarded for passing strict validation testing before arriving in customers' hands. The principles that have guided our rigorous validation practices for decades preceded a recent publication that describes five conceptual pillars for validating antibodies in an application- and context-specific manner (PMID: 27595404).
Anti-mouse MafB Recombinant Monoclonal Antibody (Rabbit, clone BLR046F) was validated by Western blot on whole cell lysate from HeLa, HEK293T, SK-N-MC, RPMI-8226, and Jurkat cells.

BioXcell
Anti-mouse CD115 (CSF1R) Monoclonal Antibody (Rat, clone AFS98) was validated by Western blot on purified mouse CD115.

Animals and other research organisms

Policy information about studies involving animals; ARRIVE guidelines recommended for reporting animal research, and Sex and Gender in Research

Laboratory animals

The following mice of the C57BL/6 background were used in this study: CD45.2 C57BL/6 wild type (WT) (The Jackson Laboratory), CD45.1 C57BL/6J WT (The Jackson Laboratory, Strain #002014), Cx3cr1Gfp/+ (The Jackson Laboratory, Strain #005582), Tmem119Cre (generated in house, see methods), Rosa26-LSL-EYFP (The Jackson Laboratory, Strain#006148),

	Cx3cr1LSL-DTR/+ (The Jackson Laboratory, Strain #025629), Ccr2-/- (The Jackson Laboratory, Strain#004999), Nr4a1-/- (The Jackson Laboratory, Strain #006187), Maffl/fl (kindly provided by Dr. Fabienne Andris), Mafbfl/fl (generated in-house, see methods), Lyz2Cre (The Jackson Laboratory, Strain #004781) and Ms4a3Cre (kindly provided by Dr. Florent Ginhoux). Myeloid-restriced Maf or Mafb depletion was achieved by crossing Maffl/fl or Mafbfl/fl mice with Lyz2Cre or Ms4a3Cre mice. A mix of male and female mice between 6 and 10 weeks of age were used for each experiment, except for chimera experiments where mice between 11 and 15 weeks of age were used. The mice were bred and housed under specific pathogen-free conditions at the GIGA Institute (Liège University, Belgium), maintained in a 12-h light-dark cycle, and had access to normal diet chow and water ad libitum. Mice were identified according to genotype and all experiments were performed with age- and sex-matched littermates. For Csf1r blocking experiments mice were randomly assigned to vehicle or isotype Ab and anti-Csf1r treatments. For experiments using IMDTR mice that were treated or not with DT, mice were randomly allocated to DT treatment or not.
Wild animals	The study did not involve wild animals.
Reporting on sex	No sex-specific differences were observed in pilot experiments. A mix of male and female mice between 6 and 10 weeks of age were used for each experiment, except for chimera experiments where mice between 11 and 15 weeks of age were used.
Field-collected samples	The study did not involve samples collected from the field.
Ethics oversight	All animal experiments described in this study were reviewed and approved by the Institutional Animal Care and Use Committee of the University of Liège (Ethical Approval #DE1956). The 'Guide for the Care and Use of Laboratory Animals,' prepared by the Institute of Laboratory Animal Resources, National Research Council, and published by the National Academy Press, as well as European and local legislations, were followed carefully. Accordingly, the temperature and relative humidity were 21°C and 45-60%, respectively.

Note that full information on the approval of the study protocol must also be provided in the manuscript.

Flow Cytometry

Plots

Confirm that:

☒ The axis labels state the marker and fluorochrome used (e.g. CD4-FITC).

☒ The axis scales are clearly visible. Include numbers along axes only for bottom left plot of group (a 'group' is an analysis of identical markers).

☒ All plots are contour plots with outliers or pseudocolor plots.

☒ A numerical value for number of cells or percentage (with statistics) is provided.

Methodology

Sample preparation	Blood was collected by retro-orbital plexus bleeding of terminally-anesthetized mice. Mice were then euthanized by cervical dislocation. Peritoneal lavage was obtained by injecting 10 mL HBSS (Lonza, Cat#BE10-508F) into the peritoneal cavity and collecting the washout. Mice were then perfused with 10 mL PBS via the left ventricle and lungs, brain, liver, spleen, intestine and colon were dissected.<br>For BM cells, femurs and tibias were dissected and cleaned of soft adhering tissue. Distal and proximal ends were opened, and BM cells were flushed out. After centrifugation, cell pellets were re-suspended in ice-cold PBS (ThermoFisher, Cat#14190094) containing 10mM of EDTA (Merck Millipore, Cat#1084181000) and cell suspensions were filtered using a cell strainer (70 µM, Corning, Cat#352350) to obtain a single cell suspension.<br>Lungs, brains, liver and spleen were cut into small pieces with razor blades, and digested for 1h at 37°C in HBSS containing 5% v/v of FBS (ThermoFisher, Cat#10270098), 1mg/mL collagenase A (Sigma, Cat#14190094) and 0.05mg/mL DNase I (Sigma, Cat#11284932001). After 45min of digestion, the suspension was flushed using a 18G needle to dissociate aggregates. Ice-cold PBS (ThermoFisher, Cat#) containing 10mM of EDTA (Merck Millipore, Cat#1084181000) was added to stop the digestion process and cell suspensions were filtered using a cell strainer (70 µM, Corning, Cat#352350).<br>Mononuclear leukocytes from lungs and livers were enriched using a Percoll density gradient (GE Healthcare, Cat#17089101) and harvesting cells from the 1.080:1.038 g/mL interface.<br>For the isolation of leukocytes from the small intestines and colons, small intestines and colons were dissected from the pylorus and the rectum, were separated from the mesenteric tissue from Peyer's patches and from fat and were placed in ice-cold HBSS with 2% FBS. Intestinal content was removed with PBS, and the small intestines and colons were opened by a longitudinal cut and washed 3 times in ice-cold HBSS with 2% FBS. To remove mucus and epithelial cells, small intestines and colons were incubated with HBSS with 2% FBS and 1 mM 1,4 dithiothreitol (DTT, Sigma, 10197777001) for 20 min with constant shaking followed by an incubation with HBSS containing 2% FBS and 1.3mM EDTA for 40 min. Tissue pieces were then cut into small pieces and incubated for 1 h at 37 ℃ with RPMI containing 2% FBS, 2 mg/mL collagenase IV (ThermoFisher, Cat#17104019) and 40 U/mL DNase I. At the end of incubation, the suspension was homogenized with a 19G syringe and filtered through a 70 µM strainer.
Instrument	Cell suspensions were analyzed with a LSRFortessa (BD Biosciences). For scRNA-seq and bulk RNA-seq, lung myeloid cells were sorted using an FACSAriaIII (BD Biosciences).
Software	Results were analyzed using FlowJo software (Tree Star Inc.).
Cell population abundance	Purity was between 90 and 95% and was determined by flow cytometry after sorting.
Gating strategy	Alveolar macrophages (AMs) were gated as Single Live CD45+ F4/80+ CD11c+ cells; Classical monocytes (cMo) were gated as Single Live CD45+ SSClo CD11b+ F4/80+ Ly6C+ CD64- cells; Patroling monocytes (pMo) were gated as Single Live CD45+ SSClo

CD11b+ F4/80+ Ly6C- CD64- cells; Bulk interstitial macrophages (Bulk IMs) were gated as Single Live CD45+ SSClo CD11b+ F4/80+ Ly6C- CD64+ cells; CD206- IMs were gated as Single Live CD45+ SSClo CD11b+ F4/80+ Ly6C- CD64+ CD206- cells; CD206+ IMs were gated as Single Live CD45+ SSClo CD11b+ F4/80+ Ly6C- CD64+ CD206+ cells; Type 1 conventional DCs (cDC1s) were gated as Single Live CD45+ CD11c+ MHC-II+ CD26+ CD64- CD172a- XCR1+ cells; Type 2 conventional DCs (cDC2s) were gated as Single Live CD45+ CD11c+ MHC-II+ CD26+ CD64- CD172a+ MAR1- cells; MAR1+ DCs were gated as Single Live CD45+ CD11c+ MHC-II+ CD26+ CD64- CD172a+ MAR1+ cells; CD64+ macrophages (CD64+ Mac) were gated as Single Live CD45+ CD11c+ MHC-II+ CD26- CD64+ CD172a+ cells; Lung neutrophils (Neu) were gated as Single Live CD45+ CD11b+ Ly6G+ cells; Lung eosinophils (Eos) were gated as Single Live CD45+ CD11b+ SiglecF+ cells; LSK were gated as Single Live Lin- Ly6A/E+ CD117+ cells; Common myeloid progenitors (CMP) were gated as Single Live Lin- CD16/32- CD117+ CD135+ CD34+ CD115- cells; Monocyte-DC progenitors (MDP) were gated as Single Live Lin- CD16/32- CD117+ CD135+ CD34+ CD115 + cells; Granulocyte-monocyte progenitors (GMP) were gated as Single Live Lin- CD16/32+ CD117+ CD135- CD34+ CD115- Ly6C- cells; Granulocyte progenitors (GP) were gated as Single Live Lin- CD16/32+ CD117+ CD135- CD34+ CD115- Ly6C+ cells; Monocyte progenitors (cMoP) were gated as Single Live Lin- CD16/32+ CD117+ CD135- CD34+ CD115+ Ly6C+ cells; Ly6C+ bone marrow monocytes (Ly6C+ BMMo) were gated as Lin- CD16/32+ CD117- CD115+ Ly6C+ cells; Common DC progenitors (CDP) were gated as Single Live Lin- CD16/32- CD117- CD135+ CD115+ CD34- Ly6C- cells; Blood cMo were gated as Single Live CD45+ CD3- CD19- Ly6G- SiglecF- CD115+ Ly6C+ cells; Blood pMo were gated as Single Live CD45+ CD3- CD19- Ly6G- SiglecF- CD115+ Ly6C- cells; Small peritoneal macrophages (SPM) were gated as Single Live CD45+ Ly6G- SiglecF- Ly6C- CD115+ CD11b+ F4/80lo cells; Large peritoneal macrophages (LPM) were gated as Single Live CD45+ Ly6G- SiglecF- Ly6C- CD115+ CD11b+ F4/80lhi cells; Kupffer cells were gated as Single Live CD45+ CD31- F4/80+ CD11bint CD64+ cells; Red pulp macrophages (RPM) were gated as Single Live Lin- F4/80+ CD11b- cells; Small intestinal lamina propria macrophages (SI LMP) were gated as Single Live CD45+ Ly6C- CD11b+ F4/80+ CD64+ cells; Colonic lamina propria macrophages (C LMP) were gated as Single Live CD45+ Ly6C- CD11b+ F4/80+ CD64+ cells; Microglia were gated as Single Live FSClo CD45int F4/80+ CD11b+ CD64+ Ly6C- cells.

For all experiments, cells were gated from debris based on FCS-A/SSC-A profile. Next, single cells were gated based on FSC-A/FSC-H profile and FCS-W/FSC-H profile. Live cells were gated as LIVE/DEAD Fixable Dead Cell- cells. . Positive populations were determined by the specific antibodies staining, which were distinct from negative populations.

☒ Tick this box to confirm that a figure exemplifying the gating strategy is provided in the Supplementary Information.
